# Senescence in Pulmonary Fibrosis: Between Aging and Exposure

**DOI:** 10.3389/fmed.2020.606462

**Published:** 2020-11-12

**Authors:** Alessandro Venosa

**Affiliations:** Department of Pharmacology and Toxicology, University of Utah College of Pharmacy, Salt Lake City, UT, United States

**Keywords:** senescence, lung fibrosis, epithelial cells, inflamm-aging, immune-senescence, aging, mesenchymal senescence

## Abstract

To date, chronic pulmonary pathologies represent the third leading cause of death in the elderly population. Evidence-based projections suggest that >65 (years old) individuals will account for approximately a quarter of the world population before the turn of the century. Genomic instability, telomere attrition, epigenetic alterations, loss of proteostasis, deregulated nutrient sensing, mitochondrial dysfunction, cellular senescence, stem cell exhaustion, and altered intercellular communication, are described as the nine “hallmarks” that govern cellular fitness. Any deviation from the normal pattern initiates a complex cascade of events culminating to a disease state. This blueprint, originally employed to describe aberrant changes in cancer cells, can be also used to describe aging and fibrosis. Pulmonary fibrosis (PF) is the result of a progressive decline in injury resolution processes stemming from endogenous (physiological decline or somatic mutations) or exogenous stress. Environmental, dietary or occupational exposure accelerates the pathogenesis of a senescent phenotype based on (1) window of exposure; (2) dose, duration, recurrence; and (3) cells type being targeted. As the lung ages, the threshold to generate an irreversibly senescent phenotype is lowered. However, we do not have sufficient knowledge to make accurate predictions. In this review, we provide an assessment of the literature that interrogates lung epithelial, mesenchymal, and immune senescence at the intersection of aging, environmental exposure and pulmonary fibrosis.

## An Introduction to Pulmonary Fibrosis

Pulmonary fibrosis (PF) is a disease of senescence, weakened anti-inflammatory activation, and aberrant resolution (1, 2). PF is a rare degenerative pathology (overall incidence of just 13–17/100,000 people/year) characterized by temporally and spatially heterogeneous injury. The excessive production and disorderly deposition of extracellular matrix proteins and collagen that accompanies disease progression is driven by (myo-)fibroblasts and immune cells clustering within aberrant alveolar structures (honeycombs) (3). PF pathogenesis and progression is unpredictable, with genetic mutation (surfactant protein B and C, mucin 5B, telomerases) and environmental factors (cigarette smoke, chronic infections) provoking functional debilitating lesions, lethal within 3–5 years of diagnosis (4, 5). Stratified epidemiological analysis clearly illustrates surge in disease incidence and prevalence in relation to age (93/100,000/year and 494/100,000/year in individuals >65 years old) (6–8). Notably, to date chronic lung pathologies represent the third leading cause of death in the elderly population (1, 9, 10). The magnitude of this healthcare problem is represented by national and global census data showing ~ =15% of the population currently over 65, and over 2 billion individuals projected to surpass that mark by the year 2050 (11–13).

As the medical field battles the COVID-19 global pandemic, scientists and clinicians have come to terms with the notion that we know very little of the mechanisms mediating lung injury and resolution in the aged and susceptible respiratory system (14–16). To overcome these limitations, this manuscript summarizes the evidence linking lung injury and remodeling in the context of biological aging and chemical exposure.

## Senescence at the Basis of Cell Dysfunction and Disease

**Figure 1:**
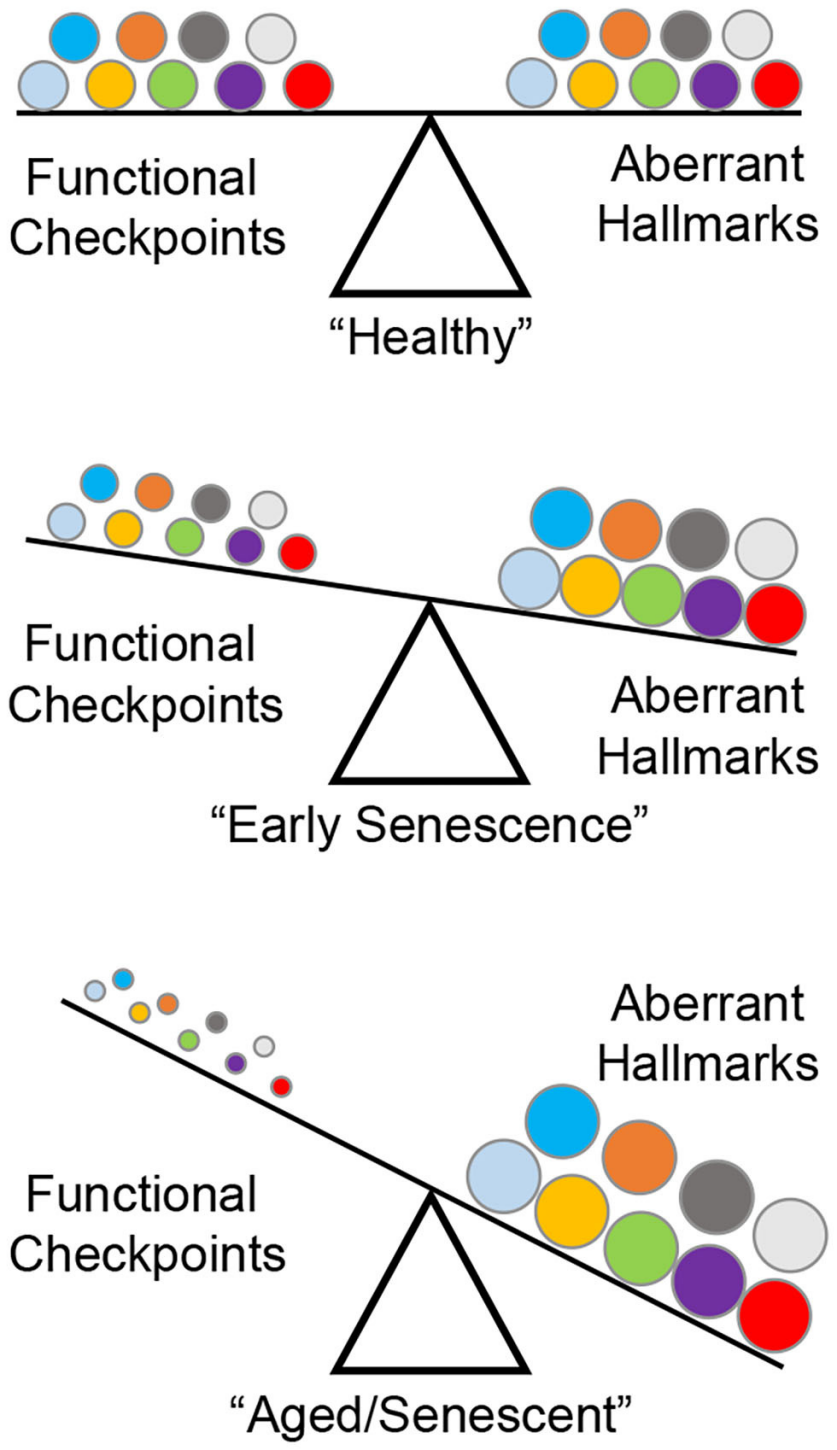
The nine “hallmarks” of cell fitness. Healthy cells rely on a balance between survival checkpoints and aberrant hallmarks. A total of nine factors have been defined: genomic instability, telomere attrition, epigenetic alterations, loss of proteostasis, deregulated nutrient sensing, mitochondrial dysfunction, cellular senescence, stem cell exhaustion, and altered intercellular communication (represented by circles in matching colors). Skewing of this equilibrium generates a progressively senescent phenotype as a result of age-related checkpoint dysfunction driving cellular toxicity.

A series of landmark reports identified nine “hallmarks” of aberrant cellular fitness shared by chronic degenerating conditions such as cancer, fibrosis, and aging: (1) genomic instability; (2) telomere attrition; (3) stem cell exhaustion; (4) epigenetic alterations; (5) loss of proteostasis; (6) deregulated nutrient sensing; (7) mitochondrial dysfunction; (8) senescence; and (9) altered intercellular communication (2, 17–23). During early life/adulthood, a checkpoint system (DNA damage response, apoptosis, unfolded protein response) ensures maximal cellular fitness (24). Aging, environmental exposure, or genetic perturbations in key functional proteins induce seemingly moderate downstream adjustments to these safeguards, but progressively edges the cell closer to developing an irreversible phenotype (Figure 1).

**Figure 2:**
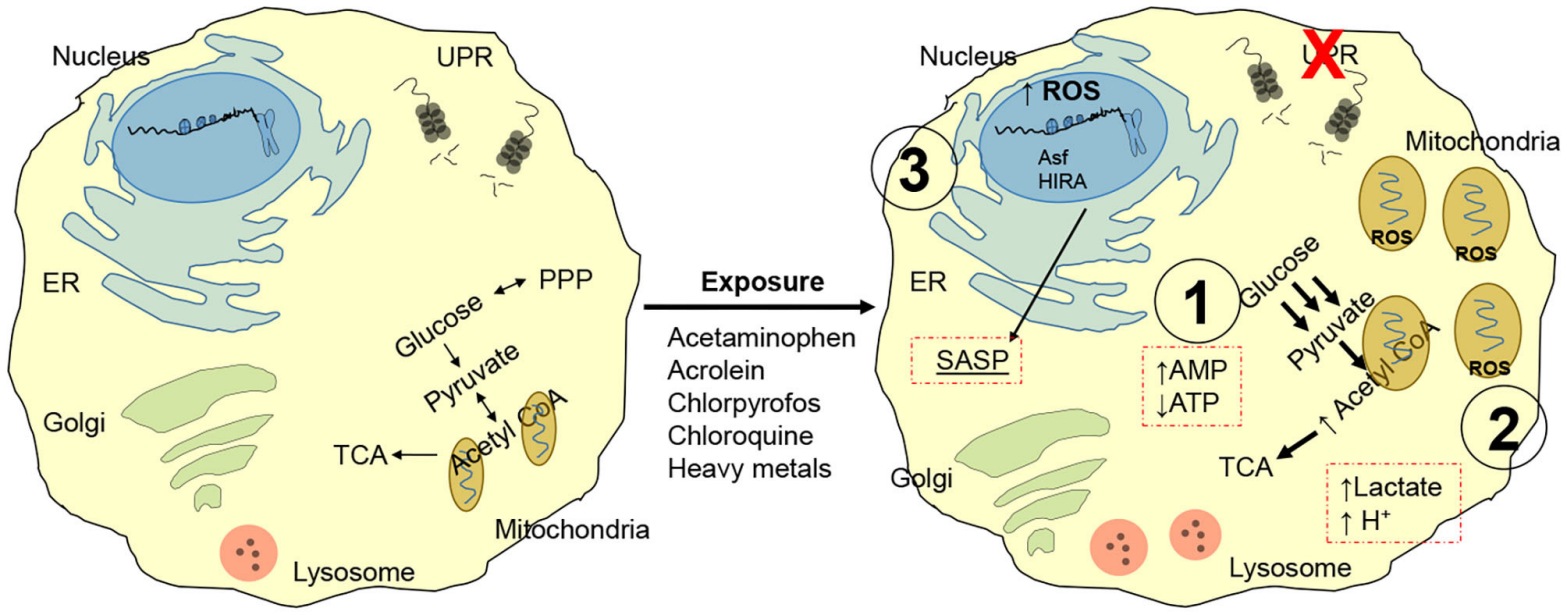
Chemical induced senescence. Acetaminophen, acrolein, chlorpyrofos, chloroquine, and heavy metals exposure disrupts unfolded protein response, UPR. Nutrient utilization is then shifted toward glycolysis, leading to: **(1)** Enhanced AMPK signaling driven by excess AMP, resulting in proliferative latency and pRB/p16 pathway activation. **(2)** Excess pyruvate enters mitochondria for conversion into AcCoA; this leads to mitochondria! overload and swelling, ROS generation and toxic cell acidification. **(3)** Oxidative stress from mitochondria damages cytoplasmic proteins and DNA; AcCoA functions as a acetyl donor for localized histone hyperacetylation, while histone chaperones Asf1 and HIRA greatly remodels chromatin independently of replicative state of the cell. Gene expression resulting from chromatin reprogram results in senescent associated secretory phenotype (SASP), enriched in tumor suppressor proteins, transcription factors, microRNAs, growth factors, proteases, and inflammatory cytokines (e.g., p-galactosidase, p16INK4a, IL-6, CXCR2, IL-1 receptor, C/EBPp, and NF-KB).

Besides the conventional notion that aging is accompanied by replication-dependent telomere erosion and defective recognition of toxic mutations, a number of convergent systemic failures contribute to the development of a dysfunctional phenotype. For instance, chemicals such as lead, nitrosamines, air/traffic pollution, carbon black are linked to telomere shortening (25, 26). Tobacco smoke and chronic ozone exposure generate reactive oxygen species and trigger mitogenesis (also linked with intracellular ROS production), thereby damaging the DNA and shortening telomeres (27). Acetaminophen, acrolein, chlorpyrofos, chloroquine and heavy metals, are all known to disrupt cellular proteostasis (i.e., unfolded protein response, UPR) (Figure 2) (28). This response is associated with a shift in nutrient utilization to favor glycolysis (and away from mitochondrial oxidative phosphorylation) (29). Due to the inefficient and slow ATP production of the glycolytic cycle, the cell progressively accumulates excess ADP and AMP and halts its capacity to proliferate, a process that requires substantial and rapidly available energy. AMP is the preferred substrate for AMPK, leading to activation of p53/p21 and pRB/p16 pathways (30). The substantial amount of pyruvate produced through glycolysis is then shuttled to the mitochondria, leading to: mitochondrial swelling; ROS generation, NADH and NADPH depletion; excess AcCoA (31–33). Mitochondrial and cellular swelling is used as a morphological biomarker to identify senescent cells. The excess ROS production promote DNA damage, which further favor the development of a dysfunctional cell and is linked to the development of a SASP (senescent associated secretory phenotype). The enzymatic conversion of pyruvate in the mitochondria generates a surplus Acetyl-CoA which is shunted to the Krebs Cycle, or in the nucleus where it is utilized as an acetyl-donors to remodel histone structure and thus, regulate cell transcription (34, 35). In support of this notion, pre-senescent and senescent cells display widespread loss of histones H3 and H4 and senescence-associated heterochromatin foci (SAHF) at the hands of the histone chaperones Asf1 and HIRA (36, 37). The role of these proteins is particular important as they represent a cell cycle independent mechanism to modify histones, a function fundamental for replication-restricted senescent cells. This epigenetic reprogram further support SASP by promoting expression and release of highly inflammatory factors including tumor suppressor proteins, transcription factors, microRNAs, growth factors, proteases and inflammatory cytokines (e.g., β-galactosidase, *p16INK4a*, IL-6, CXCR2, IL-1 receptor, C/EBPβ, and NF-KB) (38–40). SASP elicits an immense power to reshape the behavior of the surrounding tissue, to the point that just over 20% of senescent cells are sufficient to trigger systemic effects (41).

In light of this evidence, any therapeutic that effectively reduces numbers or activity of senescent cells has significant healthcare potential. While technological advancements produced a broad arsenal of pharmacological moieties, their efficacy against age- and fibrotic-related senescence has been hindered by the paucity of models that mimic the clinical course of disease (42–44).

## Chemical Exposure Accelerates Lung Senescence

The current PF paradigm proposes that repeated episodes of alveolar epithelial cell dysfunction triggered by endogenous (genetic predisposition) or exogenous stress (environmental), is necessary to trigger lung fibrogenesis. In particular, the latter remains mechanistically obscure and is often categorized as idiopathic in origin. Mutations of pivotal rheostats such as genes involved in proteostasis, telomere and mitochondrial maintenance have been abundantly mapped in the past two decades (45–51). This has led to the development of robust genetic models, including the naturally occurring senescence accelerated mouse series (SAM-1 to−8) (52), the telomerase reverse transcriptase (TERT) deficient mice (42, 43), the surfactant protein C mutant and null mice (44, 49), or through disruption of cell-cell communication via genetic modulation of key fibrotic signaling pathways (TGF-β or IL-13) (53, 54). These models all display accelerated aging, senescence and pulmonary fibrosis initiated by the lung epithelium and perpetuated by mesenchymal and immune cells (46, 55–57). By comparison, clinically relevant models of chemical induced fibrosis are limited by intrinsic differences among the thousands of environmental, dietary, or occupational stressors the lung comes into contact daily.

Reactive moieties (i.e., ozone), minerals and metals (silica, asbestos, cadmium, beryllium), wildfire and cigarette smoke, particulate matter of sizes 10 μm and below (PM2.5 and PM10), and nanoparticles/nanoplastics are widely used, yet imperfect, surrogates of chemical senescence and fibrosis (58–65). The keen observes may suggest that these models are not dependable or ineffective in predicting senescence and fibrogenesis. This is, in part, true. One clear element may be responsible for this results: age. While epidemiological evidence overwhelmingly show that age disproportionally impacts the outcome of chemical exposure and/or fibrosis (i.e., clinical PF is, on average, diagnosed at age 65), experimental modeling predominantly examines young and healthy animal cohorts. As a result, we have carefully modeled mechanistic datasets that poorly translate to aged murine cohorts, or the human condition. In support of this notion, sterile and infectious challenge (infection, radiation, and cigarette smoke) elicits heightened toxicity in aged mice compared to young ones, a response linked to the development of irreversible senescence (66–69). To overcome these limitations and provide adequate prediction of how do environmental exposures that lead to cellular senescence lead to different lung pathologies, it is absolutely necessary to test chemical exposure in aged, as well as susceptible cohorts starting, perhaps, from the aforementioned TERT and SP-C mutant mice.

## Conditions for Chemical Induced Senescence/Fibrosis

In-depth analysis of the fibrogenic effects of environmental exposure on the senescent lung is critical to advance the field and better address the needs of susceptible populations. At least three aspects need to be considered: window of exposure; dose, duration and recurrence of exposure/injury; cell type-specific responses.

### Window of Exposure

Exposure to an inhaled toxicant during early life has been suggested to increase susceptibility to disease by reshaping the parenchymal and inflammatory cell milieu (70–72). For instance, *in utero* and early life exposure to tobacco smoke, respiratory viral infections and gestational diabetes impairs lung development and function by reshaping the cellular metabolic and inflammatory machinery at the chromatin level (epigenetic), thus greatly increasing the incidence of chronic pathologies including asthma, COPD and fibrosis (73–76). Widely used as the standard model of acute lung injury, ozone represents the perfect example of an environmental toxicant that produces variable responses across the lifespan, through widespread epithelial and bronchial oxidative damage and inflammation (77, 78). The pattern recognition receptor TLR4 is partially responsible for ozone responses (63, 79). Therefore, the known age-related alteration in expression (low at birth) impacts the cellular and structural responses elicited upon exposure (80, 81).

### Dose, Duration, and Recurrence of Exposure

**Figure 3:**
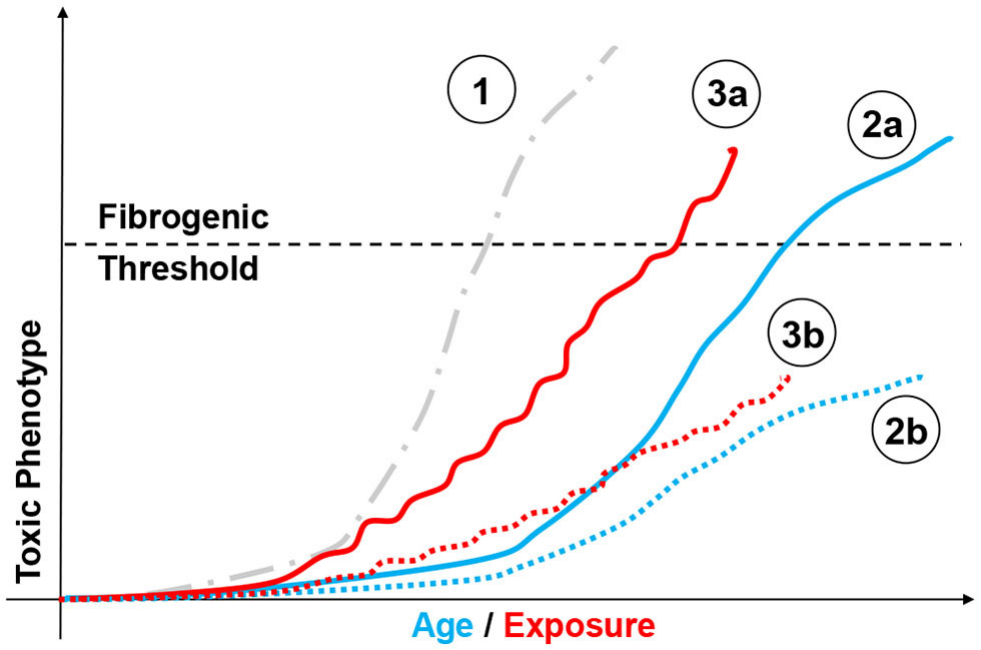
Modeling chemical exposure on the fibrotic phenotype. Depiction of possible outcomes resulting from aging and chemical exposure and their relationship to fibrogenesis. Highly fibrogenic chemical exposure (**1**, gray dotted line, 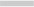) may drive rapid and possibly lethal fibrosis after a single exposure. By comparison, aging may lead to different disease profiles based on factor such as genetic instability (i.e., SP-C mutation). In such case, an individual presenting somatic mutations may be predisposed to develop a fibrotic phenotype without toxic challenge (**2a**, blue line, 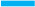), compared to a healthy individual (**2b**, blue dotted line, 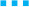). Similar responses can be observed following mild/moderate repeated exposure, with susceptible population (**3a**, red line, 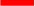) passing the “fibrogenic threshold", whereas healthy cohorts will not. (**3b**, red dotted line, 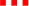) or never reach that threshold depending on factor such as genetic susceptibility. Similarly, aging may be associated with fibrotic and non-fibrotic outcomes depending on individual biological clocks.

There is extensive evidence that, although often well-tolerated, repeated toxic exposure promotes progressive genetic instability, epigenetic remodeling (i.e., cadmium); proteostatic and mitochondrial dysfunction (i.e., ozone); genesis of SASP (i.e., multi-walled nanotubes, asbestos); or all of the above (i.e., cigarette smoke and radiation) (82–87). The (often) cyclical nature of environmental/occupational exposure is also linked to exhaustion of the stem cell reservoir, their depletion, and SASP (88–90). These effects progressively diminish the ability of the lung to respond to subsequent challenges even of modest intensity. Figure 3 portrays possible outcomes resulting from to acute and chronic exposure of susceptible individuals. For instance, aging of an individual presenting somatic predisposition (i.e., SP-C mutation) may lead to fibrogenesis, compared to a health individual. Similar differences can be observed following sublethal chronic exposure, with susceptible population developing chronic pathologies. Ozone and particulate matter/dusts, once again, provide the perfect examples of moderate/sublethal and potentially recurrent stressors linked to fibrotic disease. Indeed, modeling short term acute ozone exposure produces neutrophilic, monocytic, or eosinophilic responses at doses ranging from 0.8 to 3 ppm (91–93). Repeated low-dose ozone exposure (0.8 ppm, 4 h/day, 9 days) generates subchronic multicellular inflammation with extensive airway and goblet cell involvement (94), progressing to fibrosis following 6 weeks of exposure (95). Genetic manipulation of the inflammatory collectin surfactant protein-D further supports the notion that a lifetime of sub-toxic inflammation and oxidative stress reshapes parenchymal function (senescence) and could possibly impact lung responses to exposure later in life (80, 96).

### Cell Type-Specific Responses

The lung contains more than 40 types of cells representing epithelium, interstitial connective tissue, vasculature, hematopoietic and lymphoid tissue, and the pleura (97). Each of these cell types participate to the correct function of the lung and it is therefore central to consider how chemical exposure differentially affects each cell type to better comprehend the pathogenesis of disease. For instance, mutations of the surfactant protein (SP)-A and -C, or ATP binding cassette subfamily A member 3 (ABCA3) represent well-described examples of alveolar epithelial type 2 distress resulting in chronic lung disease and fibrosis (98, 99). A number of genetic constructs successfully leveraged these mutations to produce lung fibrosis and accelerated senescence triggered by a stressed epithelium (49, 100). While to date is still unclear what effects environmental exposure elicits on such susceptible parenchyma, work from our group and others intends to fill this knowledge gap.

**Figure 4:**
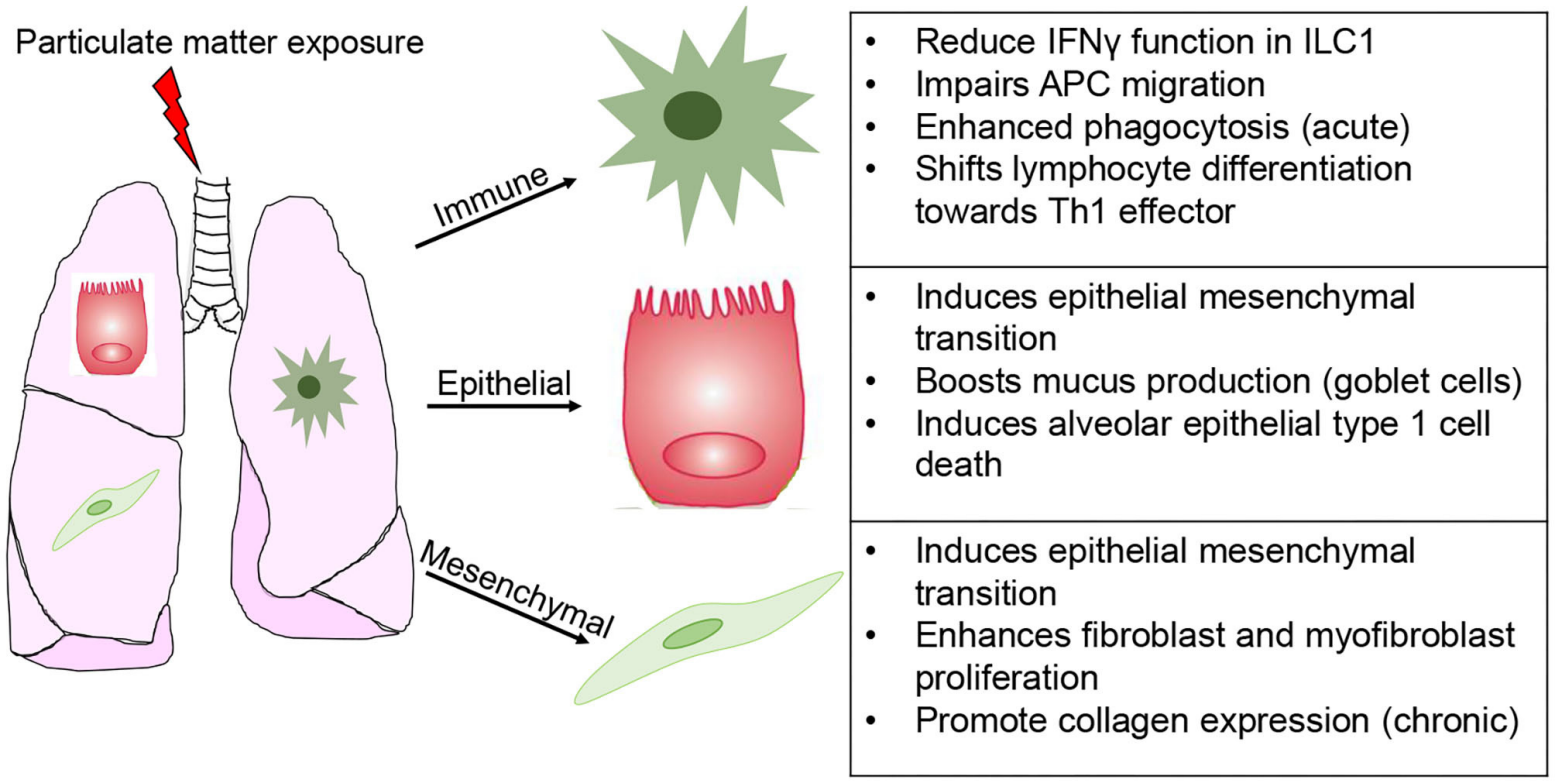
Modeling cell specific responses to chemical exposure. Simplified depiction of the divergent effects of particular matter exposure in immune, epithelial (inclusive of alveolar and mucus producing goblet cells), and mesenchymal (fibroblasts and myofibroblasts).

By comparison, chemical exposure does not promote such a cell specific response, often leading to varying degrees of stress, ranging from susceptibility to disease later in life to irreversible senescence and pathogenesis of disease (101, 102). Particulate matter exposure has been widely studied for its importance in lung health (Figure 4). This environmental and occupational mixture is known to induce ROS production, activating the inflammasome pathways and triggers unfolded protein response in lung epithelial cells (103). Depending on size (2.5 or 10 μm), dose, and duration of exposure PM elicits epithelial cell death (acute), as well as exacerbation of chronic pulmonary conditions (asthma and COPD) and epithelial to mesenchymal transition (104). Based in these responses it is unsurprising that PM promote fibroblast and myofibroblast proliferation, a response fundamental for fibrogenesis (105–107). At the levels of mucus producing cells (goblet cells), PM results in MUC5B hypersecretion (103). Furthermore, PM engages and functionally impacts innate like cells type 1 (ILC1), thereby blunting their interferon gamma (IFN-γ) production and cytotoxic function; induces antigen-presenting cell-mediated inflammatory responses, while impairing their migration; enhanced neutrophil and eosinophil responses; and shifts lymphocyte differentiation toward an effector phenotype (Th1-like) (108–111). Lastly, the effects on endothelial function (disruption of tight junctions) have significant repercussion on the susceptibility to cardiovascular disease and infarction (112–115). Notably, modeling the toxicity of PM is further complicated by the fact that intrinsic composition (levels of metals, polycyclic aromatic hydrocarbons, carbon black content) and secondary chemicals being carried (LPS and allergens) ranges across time of the year and location where it is collected (116–118). In what seems like a prohibitive task (investigating the effects of hundreds of chemically diverse moieties on dozens of cell types), technological advancements in single cell sequencing analysis and multi-omics approaches significantly eased these challenges. The next section will summarize epithelial, mesenchymal and immune cell senescence induced by aging or chemical exposure.

## Cellular Responses in Senescence and Fibrosis

### Lung Epithelium

While it is well-established that initial respiratory functions are achieved in the nasal epithelium and upper airways, the largest share of research is devoted to the study of alveolar type-1 pneumocytes (AT1) due to their central role in gas exchange. A second alveolar epithelial population, termed AT2, has gained broad recognition as a multipurpose unit in charge of pulmonary surfactant production; regulation of fibroblast proliferation; communication with resident immune cells during homeostasis and injury; control of vascular endothelium permeability to peripheral leukocytes; and replenishment of damaged AT1 cell and mesenchymal (epithelial-mesenchymal transition) pool in stressful conditions (119–121). It is therefore unsurprising that epithelial cell dysfunction produces such a multifaceted phenotype, ultimately linked to senescence and lung remodeling. Supported by clinical evidence, TERT and surfactant protein-C mutant lines today represent robust platforms for modeling epithelium-driven fibrosis (44, 49, 50, 122–124). Mutations in the cystic fibrosis-linked ubiquitin ligase NEDD4-2 produces aberrant epithelial Na+ channel (ENaC) and pro-SP-C localization, processing and degradation, leading to airway surface liquid depletion, impaired clearance of inhaled irritants and progressive architectural and functional alterations consistent with cystic fibrosis-like disease (44, 49, 50, 122–125). The tremendous influence that these proteins elicit on epithelial cell survival and proliferation prioritizes their evaluation in the context of aging. While not directly examining the role of SP-C mutations, experimental evidence linked age-related senescence of the surfactant protein machinery to poor survival following sublethal bacterial challenge (LPS) (126). Similarly, there is clinical evidence that TERT mutations, both familial or exposure-induced (i.e., cigarette smoking), is accompanied by premature deaths (127). The paucity of data that comprehensively examines the responses of a dysfunctional lung epithelium to environmental challenge blurs our ability to determine the mechanistic overlap preceding fibrogenesis. To the same point, chemical challenge has been predominantly examined in juvenile/healthy lungs. While these studies helped defining senescent-like phenotype following exposure, a conspicuous scientific gap remains.

At its essence, the most complex element associated with the study of aging, chemical exposure, or fibrosis, is the asynchronous nature of cellular senescence that accompanies pathogenesis of the phenotype. Application of diffusion pseudotime, a computational single-cell method that can trace the dynamics of biological processes and predict cell fate, may elegantly address this problem (128). Recent use of this methodology to study the evolution of the epithelial cell milieu during bleomycin induced lung fibrosis identified a unique transitional stem cell state, defined by *Krt8* expression, involved in regeneration and healing (129). Lineage tracing techniques and human-derived organoid cultures recently identified a similar population of AT2 cells on their way to terminally mature AT1 cells (130, 131), while single-cell transcriptomics of human IPF and COPD lungs have morphologically (termed basaloid cells for their distinct non-AT1/squamous non-AT2/cuboidal structure) and transcriptionally linked this regenerative subset to fibrotic remodeling (132). While investigation of these transitional epithelial cells is still in its infancy, it is tempting to propose their involvement in resolution of chemical-induced injury; their progressive dysfunction in the context of aging; and to ask whether repeated stress can “exhaust” their replicative potential (133). Addressing each of these questions may advance out understanding of senescence.

A number of recent reports have provided a considerable foundation on the biology of the aging lung epithelium. By combining single-cell RNA-sequencing analysis and proteomics, Angelidis and colleagues generated a comprehensive cell-type specific atlas of the 3 and 24 month old murine lungs (unchallenged), and convincingly presented both ultrastructural and functional changes associated with senescence, including extracellular matrix deposition and epithelial inflamm-aging (134). These epithelial changes reflect, at least in part, organ level dysfunction of the aging lung, characterized by reduced mechanical tissue remodeling, aberrant alveolar derecruitment and impaired oxygen saturation (44, 135). The fibrotic lung presents comparable epithelial and organ wide (functional) alterations, oftentimes in an accelerated and more widespread fashion. The heterogeneity of the injury also complicates assessment of epithelial senescence from a whole organ perspective. As discussed above, instability generated by TERT, ABCA3, SP-A, and SP-C mutations or fibrogenic exposure is accompanied by an epithelial phenotype that aligns with age-induced senescence (cell cycle checkpoint disruption and SASP) (136–138). To date, only correlative evidence (epidemiological) links ambient pollution exposure to acute inflammatory exacerbations that rapidly accelerate lung function decline in IPF patients (102, 139, 140). By combining this observational evidence with the currently available pulmonary disease models, the next decade of pulmonary research has important clinical implications to our pursuit of mechanistic answers of sublethal toxicity in the susceptible lung.

### Mesenchymal Cells: Fibroblasts and Stromal Cells

Fibroblasts are highly proliferative cells crucial in maintaining alveolar structural integrity and architecture during homeostasis and throughout the injury resolution process. Understanding the biology of fibroblast senescence is central to link aging and exposure to chronic lung pathologies (141), as their uncontrolled proliferation and/or senescence causes aberrant alveolar remodeling (i.e., impaired gas exchange function). Fibroblast senescence can be triggered directly (somatic mutations and environmental stress), or indirectly (TSLP, IL-25, and IL-33 rich milieu produced by neighboring senescent cells) (142–144).

Aging significantly impacts the extent of fibroblast senescence. For instance, toxic challenge of young primary fibroblasts triggers a “reversible” senescent state, still capable of eliciting programmed cell death mechanisms or resolution pathways. By comparison, cells from aged mice undergo myofibroblast trans-differentiation and develop a profibrotic phenotype (145–147). Analysis of accelerated senescence models (TERT and SAM1-8 murine lines) or human IPF fibroblasts demonstrate aberrant replicative responses (increased expression of cyclins, renin-angiotensin peptides, insulin-like growth factor–binding proteins 3 and 5, Wnt signaling pathway), and altered survival signaling (i.e., apoptosis and autophagic flux) (148–151). A number of environmental stressors, such as ozone and particulate matter, are known to trigger similar oxidative stress and survival pathways. While environmental/occupational exposure to these chemicals is often time subtoxic, chronicity and window of exposure progressively burdens healthy fibroblasts to develop a senescent phenotype (151, 152). This notion is corroborated by experimental modeling using “high-impact” fibrogenic stressors such as bleomycin and gamma radiation, which produces a senescent phenotype comparable to that described in aging (aberrant proliferation and survival, as well as MCP-1, PAI1, TNF-α, MMP10, MMP12, Col1a1, TGFβ, p16, and p53 overexpression) (153).

Mesenchymal Stromal/Stem Cells (MSC): For the past 20 years the acronym MSC has been used to define several subsets belonging to the same mesenchymal family (mesenchymal stem cell, mesenchymal stromal cell, and multipotent stromal cell). Today, it defines multipotent mesenchymal stromal cells, a nomenclature that separates them from mesenchymal stem cells on the basis of self-renewal and the capacity to differentiate down multiple lineages. While there is no truly unique MSC marker, a combination of hematopoietic progenitor markers CD73, CD90, and CD105 appears to be well accepted (154). This population is canonically associated with a bone marrow origin, but it has been reported that lung resident MSCs are involved in local sustenance of the mesenchymal compartment (155). Although still debated, some evidence also suggest that lung MSCs might be susceptible to differentiate into myofibroblasts and promote airway fibrosis (156).

Dampened stromal signaling in aging immune organs such bone marrow, thymus, lymph node, and spleen is thought to be responsible for the progressive contraction in lymphoid cell numbers and loss in adaptive immune system function in the elderly population (157). The inflammatory microenvironment described with age (inflamm-aging) also triggers stromal cells to produce activation factors (TNFα IL-1β, IL-6, MCP1, MMP-12, MMP-13) that contribute to the phenotypic reprogramming of peripheral myeloid cells prior to their egression to the lung (1, 158, 159). Chemical exposure to pesticides, doxorubicin, bleomycin and radiation demonstrate that the added pressure provided by acute or chronic exposure accelerates stromal cell senescence, thereby contributing to the pathogenesis and progression of the fibrotic response (153, 160, 161). Indeed, primary bone marrow MSCs from IPF demonstrate a highly senescent phenotype, characterized by mitochondrial dysfunction, debilitating DNA damage, and the secretory capacity to induce senescence in normal fibroblasts (162).

### Immune Cells

Myeloid and lymphoid cell senescence can be described as a combination of heightened inflammatory tone in homeostatic conditions (inflamm-aging), and abnormal immune reaction following challenge (immune-senescence) (163, 164). These responses result in reduced pathogen clearance and inflammatory resolution at the innate immune level. By comparison, senescence of the adaptive system is associated with blunted humoral response and failure to recognize “self” and increases the susceptibility to develop autoimmune disorders. As we dive into cell specific mechanisms of senescence, it is important to define its divergence from “exhaustion,” the altered differentiation state observed in chronic infection and cancer. Granted, these two states share a number of features inherent to the function of key transcription factors, metabolic derangement, and a failure to transition to quiescence. However, proliferative dysfunction (irreversible in senescence) and activation state (incompetent in exhausted T cells) is profoundly different (165, 166). Justification of these differences may provide important insights to our understanding of both cellular conditions.

As introduced in the previous sections, senescence is triggered by biological aging and accelerated by fibrotic remodeling, whether induced by somatic mutations or secondary to external challenges (167, 168). Aberrant activation of the immune system has profound effects on disease outcome. To counter excess immune activation in pulmonary injury and fibrosis we adopted broad spectrum immunomodulation (corticosteroids, anti-cytokine antibodies) (169, 170). However, its efficacy has been sporadic and a number of dilemmas remain, as to whether steroid immunomodulation is at all effective in toning down the activation of a senescent immune cell; or if the doses used to treat elderly individuals are adequate to address the myeloid lineage expansion typical of aging (171, 172).

The ever-increasing toolbox of immunological models available to investigate immune cells in pulmonary fibrosis have rarely been used to examine aging and senescence. These include, germ line and inducible knock outs targeting M-CSF, GM-CSF, CD68, and CCR2, as well as Cre/Lox and diphtheria toxin depletion lines targeted against CD25^+^, LysM^+^, CD11b^+^, and CD11c^+^ cells (93, 173–177). Adoptive transfer, bone marrow chimerism, parabiosis, and lineage tracing have also provided substantial data on inflammatory cell dynamics in lung injury (178, 179). The advent of CRISPR/Cas9 technology has expanded even further the range of possibilities at hand to investigate these questions (180, 181).

It is fundamental to recognize that the behavior of immune cell subtypes is unique and often dependent on the surrounding environment. The failure of broad-spectrum immunomodulatory therapy in PF is a reminder of this. The next section summarizes the current knowledge of age- and chemical-induced senescence on a cell by cell basis.

### Macrophages (an *in vitro* Preamble)

Macrophages are considered the archetypal resident guardians of any tissue, acting in unison with their neighboring cells to mount the adequate response to challenge. Today's deep understanding of macrophage phenotype and function results from extensive *in vitro* testing of immortalized cell-lines and bone marrow-derived macrophages to generate two phenotypic states: M1/pro-inflammatory (classically activated; elicited using IFNγ or LPS) and M2/anti-inflammatory (alternatively activated; induced by IL-4/13 or IL-10) (Table 1) (182). This research provided the necessary insights to define macrophage extreme plasticity and a bench work for comparison against *in vivo* responses. While *in vitro* assessment of aging is prohibitive, extrapolation of the effects of age-related senescence on macrophage function demonstrated positive results. Utilizing analogous stimulatory conditions (M1: LPS or IFNγ; M2: IL-4) evidence show that macrophage isolated from old mice and conditioned with M1 or M2 prototypical activators exhibit blunted activation compared to macrophages isolated from young mice (iNOS, IL-6, TNFα, and IL-1β, as well as YM-1 and Arginase), a response consistent with the notion of immune-senescence (198). Similarly, bone marrow derived macrophages isolated from p16 knock out mice demonstrate a phenotype resembling that of IL-4 treated M2 cells and inability to elicit pro-inflammatory functions upon IFNγ challenge (199). This wealth of information provided the foundation of *in vivo* research on fibrosis, senescence and aging, and emphasized the perception that macrophage phenotype and activation is organ-specific and non-dichotomous (200, 201).

**Table 1:** Phenotypic characterization of nine prototypical macrophage populations including M1, M2a/b/c/d, Mox, Mhb, and M4.

**Phenotype**	**Trigger**	**Transcription Factor**	**Function**	**Activation signature**
M1	IFN-γ, TNF-α, and LPS	STAT1/5, IRFs, NF-κB and AP-1	Antibacterial, Destructive, Th1 immunity; Type-IV hypersensitivity, tumor resistance	TLR-2/4, CD80, CD86, iNOS, and MHC-II on the surface. Produce TNF-α, IL-1α, IL-1β, IL-6, IL-12, IL-23, CXCL9, CXCL10, CXCL11
M2a	IL-4/-13	STAT3/6, glucocorticoid receptor, (PPAR)-γ and -δ, STAT6, IRF4, JMJD3	Repair and remodeling (pro-fibrotic); Th2 immunity; endocytic activity; cell growth	YM1, FIZZ1, Arg-1, CD206, IL1R surface expression. Produce IL-10, TGF-β, CCL17, CCL18, and CCL22
M2b	TLR ligands + IL-1β		Th2 immunity; Immunoregulatory (breadth and depth of inflammatory responses)	CCL1, TNF-α, IL-1β, IL-6, and IL-10
M2c	Glucocorticoids, IL-10 and TGF-β		Immunoregulation, tissue repair, matrix remodeling; Clearance of apoptotic tissue	TLR-1/8, Arg-1, CD163, CD206 surface expression. Produce IL-10, TGF-β, CXCL13, CCL16, and CCL18
M2d	Adenosine + TLR2/4/7 antagonists		Pro-angiogenic; clearance of apoptotic tissue	IL-10R, IL-12R surface expression; no Dectin-1 expression. Produce VEGF, IL-10 and iNOS; low levels of TNF-α and IL-12; intermediate Arg-1
Mox	Oxidized phospholipids	Nuclear factor erythroid 2–related factor 2 (NRF2), Nurr1	Pro-atherogenic. Reduced phagocytic and chemotactic function	TLR-2 surface expression. Produce NRF2 response genes, reactive oxygen species, IL-1β and IL-10
Mhb	Haptoglobin	–	Phagocytic (erythrocyte clearance)	HO-1 and CD163 surface expression
M4	CXCL4	KLF2	Pro-atherogenic; no phagocytic capacity	No CD163, MHC-II, and HO-1 expression. Produce TNF-α, IL-6, CCL2, CXCL8, MMP-12, and S100A8
**References**: (182–197)				

*Table describes the chemical mixture necessary to trigger phenotype in vitro; transcription factors involved in macrophage activation; biological function; and activation signature. Appropriate references are listed at the bottom of the table*.

### Circulating Monocytes

Peripheral monocytes (Mo) can be simplistically defined as immature myeloid intermediates recruited to sites of injury through chemical gradient. Three major populations of circulating monocytes have been recognized both in mice (CD11b^+^CD11c^−^Ly6C) and humans (CD11b^+^HLA^−^DR^+^CD169^−^): classical monocytes (CCR2^+^Gr1^−^Ly6C^hi^ in mice; CD206^−^CD14^++^CD16^−^ in humans) are short lived (mean survival ≈1 day), acting as a reservoir to replenish tissue resident cells following injury. Notably, restoking the resident macrophage compartment was recently shown to produce a highly fibrogenic monocyte-derived subtype in experimental models of primary allograft rejection, rheumatoid arthritis, and fibrosis (100, 179, 202–204). Intermediate monocytes (mean survival ≈4 days) patrol the vasculature and are known to transition to non-classical monocytes. Non-classical/intravascular (CX_3_CR1^+^Gr1^−^Ly6C^lo^ in mice/CD206^+^ CD14^+^CD16^+^ in humans) represent subset known to patrol the endovascular space by tightly adhering to the microvasculature (205–208). These cells survive up to 7 days in the circulation and replenishing the interstitial and alveolar compartment in conditions of stress, and playing important role in inflammatory termination and tissue remodeling (179, 209).

The limited invasiveness of human blood monocyte collection and the simplicity of phenotypical characterization on the basis of surface expression has provided a useful tool to interrogate their role in aging and pulmonary disease. Although aging does not affect total monocyte counts, non-classical CD14^+^CD16^+^ monocytes significantly increased with age, but display reduced HLA-DR (aka MHC-II) and CX_3_CR1 surface expression in the elderly; by comparison, classical CD14^++^CD16^−^ monocyte numbers are not affected by age (210, 211). In the context of pulmonary disease (fibrosis and COPD), number of CX_3_CR1^+^ anti-inflammatory non-classical monocytes are inversely proportional to disease severity; this is juxtaposed to the increases in classical CD14^+^ and CCR2^+^ monocytes in patients with poor prognosis (212–214). Evidence of monocyte functional senescence is demonstrated by analysis of blood monocytes collected from aged cohorts present blunted responses to bacterial infection (LPS) as a result of decrease in TLR1 and TLR4 surface expression (215), as well as substantial age-associated defect in CD80 expression and functional engagement (89, 216). With blood derived monocytes are clinically (easily) accessible, it is surprising to see such limited number of datasets screening for transcriptional changes across an individual lifespan in conjunction with the progression of fibrosis. It would be a tremendous achievement to direct research efforts onto this question and combine it with the current advancements in therapeutics aimed at control myeloid cell phenotype (217–219).

### Alveolar and Interstitial Macrophages

In terms of pulmonary immunobiology, alveolar macrophages (AMs) represent the most studied population. These long-lived resident sentinels constantly communicate with the surrounding parenchyma to elicit innate and adaptive immune activities, promote immune-tolerance, and participate in lung surfactant reuptake (209, 220, 221). One notable immunological discrepancy between the murine and human lung is represented by the frequency of AMs. Westphalen et al., estimated one macrophage per three alveoli (222). By comparison, the human lung is composed by ~480 million alveoli and 2.1 billion alveolar macrophages, thus suggesting a ratio of over four macrophages/alveolus (223, 224). The evaluation of human alveolar macrophage ontogeny can only be inferred from murine evidence (225). The advent of fate mapping approaches defined fetal liver origin for murine AMs, while attributing their homeostatic sustenance throughout life to local proliferation (209, 226–228). These cells can be characterized by a unique signature of transmembrane integrins and sugar binding lectins associated with maturity (MerTK^+^F4/80^+^SigF^+^Ly6C^lo^CD64^hi^). Consistent with this notion, human AMs can be defined by their expression of CD11b^+^HLA-DR^++^CD206^++^CD169^+^ (225, 229, 230).

A second resident macrophage population is represented by interstitial macrophages (IMs). These cells originate from sequentially recruited progenitors in the yolk sac and bone marrow, an observation that suggests functional diversity across resident lung macrophages on the basis of ontogeny (227). CX_3_CR1 reporter mice has helped visualization of IMs within the bronchial parenchyma, in the proximity of the lymphatic vessels. Flow cytometric characterization of IMs reveals strong similarities to blood monocyte's in mice and humans (mouse IMs: MerTK^+^CX_3_CR1^+^F4/80^+^SigF^−^Ly6C^lo^CD64^hi^ and monocytes: MerTK^−^F4/80^+^SigF^−^Ly6C^lo^CD64^int^; human IMs: CD11b^+^HLA-DR^++^CD206^+^CD169^−^; and monocytes: CD11b^+^HLA^−^DR^+^CD206^−−/+^CD169^−^), thus indicating blood monocytes replenish the interstitial compartment across the lifespan (227, 231–234). Unbiased (single-cell) and biased (cell sorting) RNA-sequencing was utilized to functionally define unique IM subpopulations. The resulting datasets independently found Lyve-1 and MHC-II as discriminants. While providing nuanced differences, these studies demonstrated: (1) heterogeneity within the interstitial compartment; (2) transcriptome divergence from alveolar macrophages; (3) existence of parallel populations in heart, fat, and dermis; (4) monocytic origins (Ly6C^hi^); (5) mobilization/residency to specific tissue sites, wither adjacent to nerve bundles (Lyve1^lo^MHCII^hi^) or blood vessels (Lyve1^hi^MHCII^lo^); (6) immunomodulatory functions (their depletion exacerbates fibrosis following challenge) (231, 235, 236).

A third population of resident macrophages is represented by monocyte-derived alveolar macrophages (MoAMs). This population develops in response to significant challenge of the lung, sufficient to recruit peripheral monocytes. These immature myeloid cells have been shown to mature into macrophages, replenish the alveolar compartment, and persist in the tissue for extended periods after bleomycin induced injury, where they elicit a fibrotic phenotype (100, 179, 237).

Senescence of the resident macrophage compartment has been investigated across the spectrum of lung health (genetic susceptibility, acute and chronic chemical challenge, and biological aging), but seldom in combination. Exposure to the warfare agent, nitrogen mustard is accompanied by early pro-inflammatory macrophage activation, followed by transition to a pro-resolution/pro-fibrotic phenotype (238). While identification of a senescent phenotype was beyond the scope of these studies, RNA-sequencing analysis of lung macrophages at a time coordinated with fibrosis found features consistent with senescence, including apoptosis, p53, and cell cycle signaling, paired with morphologically aberrant appearance (foamy) (239, 240). In the context of biological aging, tissue-resident macrophages persist in the lung without input from bone marrow-derived monocytes. Aged alveolar macrophages demonstrate increased signs of inflamm-aging (interferon signaling) and down-regulated cell cycle signaling, phagocytotic and antigen recognition function (241, 242). Preliminary evidence by McQuattie-Pimentel et al., elegantly shows that resident macrophages from aged cohorts adoptively transferred to young lungs acquires a transcriptome prolife reflecting the age of the recipient, thus suggesting that lung microenvironment governs macrophage behavior (243). While the work is still in preprint form, the authors bring up significant points related to the differential responses of lung resident AMs and MoAMs to a second challenge. This (and other) work could reshape how we view macrophage biology. A more complete understanding of monocyte biology in the context of aging and fibrosis could help identify unique signatures to define monocyte-derived alveolar macrophages, and even achieve selective targeting during the fibrogenic process.

### Eosinophils

Eosinophils are extensively studied in the context of eosinophilic esophagitis, hyper-eosinophilic syndrome, asthma, allergy, and parasitic infection. There is observational evidence linking eosinophils to fibrosis and COPD; however, not much has been done to show their functional role (244–249). Eosinophils perform a number of functions important in tissue remodeling, including modulating lymphocyte recruitment and homeostasis and coordinating Th2 polarization (250). As seen with macrophages, ontogeny impacts cell behavior and physiological function. Indeed, resident eosinophils (SigF^int^CD62L^+^CD101^lo^) have been reported to reside within the lung airspace where they display a regulatory phenotype, while bone marrow cells exhibit a highly destructive phenotype (IL-5 dependent, SigF^hi^CD62L^−^CD101^hi^) (251, 252).

Eosinophilia is infrequent and not well-understood in pulmonary fibrosis. Nevertheless, blood eosinophil counts have been shown to be a valuable biomarker predicting development of acute inflammatory exacerbations and prognosis in chronic pulmonary disease (253–255). These intermittent events are currently treated with corticosteroids, although a number of large clinical trials demonstrate that prednisone has minimal positive impact (and could even be detrimental) on patient survival during or after acute inflammatory exacerbations (256–258). Asthma therapy provides important data on the pro-apoptotic effects of broad spectrum corticosteroid therapy (prednisone, dexamethasone) (259). In the absence of stratified analysis to determine whether eosinophilic IPF patients represent the group most responsive to steroid therapy, it is tempting to argue that eosinophil blood count could provide a valid aid to improve therapeutic regimen and, perhaps, disease outcome. One caveat could be related to age-related diminution in peripheral blood eosinophil counts and function (reduced IL-5 stimulation) across a lifespan, since the median age of PF diagnosis is ≥65 and by this time eosinophil development from bone marrow progenitors is significantly reduced (260–262). This could, in turn, reduce steroids responsiveness in the elderly to a level comparable to that seen in asthma and COPD individuals resistant to glucocorticoid therapy (262–266). In this context, it is interesting that parabiosis or adoptive transfer attempting to replenish old mice with juvenile/younger eosinophils successfully prevented age-related declines in physical and immunological functions (267).

### Dendritic Cells

Dendritic cell (DC) represent a cellular link between innate and adaptive immunity. Their ever-expanding taxonomy reflects their myeloid/lymphoid lineage differentiation as conventional DCs (CD103^+^CD11b^+^CD11c^+^MHC-II^+^), plasmacytoid DCs (CD11c^+^PDCA1^+^B220^+^), monocyte-derived DCs (CD11b^+^CD141^+^) (268). DCs are primarily tasked to perform antigen recognition functions. Their age-related senescence has been associated with increased immune response against self-antigens (269). This effect, combined with diminished secretion of innate cytokines such as type I and III interferons by plasmacytoid DCs, as well as reduced expression of the anti-inflammatory cytokine, IL-10, significantly impacts the principal functions of these cells with respect to impaired vaccine responses in the elderly (270). It is currently unclear whether DC's functional senescence results from permanent remodeling of the epigenome leading to aberrant response to challenge, and identification of inaccessible DNA regions during the inflammatory response is an active area of DC research (via ATAC-sequencing analysis alone and/or in combination with single-cell RNA-sequencing) (271).

Dendritic cell presence in the lung is dependent on bone marrow recruitment of pre-DCs, that mature locally into DC subsets (272). Fate-mapping analysis shows that CD103^+^ cells arise almost exclusively from common DC progenitors (maturing from macrophage-DC progenitors), while that number drops drastically for CD11b^+^ DCs (273). Plasmacytoid DCs are critically dependent on IRF8 and STAT3 signaling, while GM-CSF and signaling via STAT5 inhibit their maturation (274). It is therefore unsurprising that STAT3 signaling declines with aging, a response that may explain the systemic contraction of this DC subset (275, 276). The role of lung resident dendritic cells in pulmonary fibrosis remains largely unstudied. Single cell sequencing analysis of IPF lungs shows only minimal increases in dendritic cell population (132), while experimental modeling (adenoviral-TGFβ overexpression) indicates that fibrogenesis is accompanied by increases in CD11b^+^ and CD103^+^ DCs, but that their ablation does not affect lung remodeling (277).

### B Cells

The lung adaptive/humoral immune response is carried by highly specialized lymphoid populations, including B cells and T cells. There are some important differences between humans and mice. Much of the information on B cell cellular and molecular pathways described here was derived in murine models. Canonical B cells are mostly known for their adaptive (immunoglobulin-dependent) function against infections and cancer (278). CD19^+^ B cells populate ~5% of the lung immune compartment, and their numbers almost quadruples in non-small cell lung cancer (279). Two self-renewing B cell subtypes, B1A and B1B, have been described to develop in the fetal liver from a distinct progenitor and perform innate-like functions in the lung (280). These subsets can be distinguished by their differential expression of CD5 and CD43. They mobilize from the bone marrow to the respiratory tract in a CXCL13 dependent fashion (281), and trigger an IgM and IgA response following IL-5, IL-10, and TLR-agonist signaling (282). Their role in aging and fibrotic senescence is not well-established, but mounting evidence suggests that fetal exposure may produce a battery of specialized subtypes that favor pathogenesis and progression of lupus, diabetes and asthma (283–285).

Evaluation of telomere length as a proxy for senescence is abnormal in B cells since naïve and germinal center B cells exhibit long telomeres, while circulating and memory B cells show extremely short ones (286). By comparison, B cells functional senescence is more linear, as demonstrated by increased incidence of infection in the elderly (287, 288). Decline in bone marrow stromal cell IL-7 production significantly impacts B cell numbers and their maturation. As a result, a highly autoreactive and pro-inflammatory (inflamm-aging) senescent B cell is noted (287–290). Mechanistically, this response appears to be driven by progressive reliance on inflammatory TLR7/TLR9 engagement, rather than the B cell receptor, to trigger a humoral response (287, 291–293). In turn, these autoantibodies lead to chronic lung lesions that can be only resolved via scarring/fibrosis. Perhaps as a compensatory mechanism, aberrant expansion of ectopic inducible bronchus-associated tissues (iBALT) independently supports the maturation and selection of B cells (CD5^+^ and CD20^+^) in aged, COPD, and IPF clinical cohorts, in particular those with cigarette smoking history (294–296). Single cell sequencing and proteomic analysis of lungs and peripheral blood cells from IPF individuals shows strong evidence of B1 and CD38^+^CD138^+^ plasma cells accumulation, while experimental modeling of fibrosis generated strong correlation between the degree of pulmonary fibrosis and B-cell numbers in the germinal center (297–300). The involvement of B cells in sterile (environmental) injury is not as well-established, aside from allergen induced IgE production in aging asthma cohorts (301). Nevertheless, the potential role of memory and B1 B cells in shaping humoral immunity against senescent cells should not be overlooked (302, 303).

### T Cells

There are several non-lung resident T cell subsets defined by their surface expression and cytokine production (Table 2). These cells reside in primary and secondary lymphoid organs (bone marrow, spleen, thymus, lymph nodes), and can be promptly mobilized to the lung through the lymphatic and vascular system. By comparison, only a handful of lymphocyte subsets are recognized as “lung resident,” with their appearance conditional to pathogen exposure across the lifespan (i.e., flu) (311–314). These include tissue-resident memory T cells (TRM), innate (innate lymphoid cells, ILCs), and unconventional T cells (invariant natural killer T cells, iNKT; CD8αα^+^ cells; mucosal-associated invariant T cells, MAIT; γδ T cells; and intestinal intraepithelial lymphocytes, IELs) (304, 305). Our understanding of tissue resident lymphocyte senescence is limited by subset novelty or their abundance (TRMs expressing a specific antigen are infrequent). Nevertheless, there are numerous reports describing senescence of the machinery in charge of T cell differentiation and selection as well dysfunction of peripheral lymphocyte populations due to toxic microenvironment. Due to the abundance of T cell subtypes to be discussed, a single section would not be sufficient. There are comprehensive reviews that highlight T cell senescence and human health (315–317).

**Table 2:** Phenotypic characterization of peripheral and tissue resident lymphocyte populations including CD4, CD8, Th17, Treg, innate (innate lymphoid cells, ILCs), and unconventional T cells (invariant natural killer T cells, iNKT); CD8αα^+^ cells; mucosal-associated invariant T cells, MAIT; γδ T cells; and intestinal intraepithelial lymphocytes, IELs).

**Phenotype**	**Trigger**	**Transcription Factor**	**Function**	**Activation signature**
CD4^+^ (Cellular Response)	Antigen presenting cell and epithelial signals (CXCL10 and CXCR3)	STAT4, Tbet	Th 1 Immunity (Enhance macrophage killing activity, proliferation of cytotoxic CD8^+^ T cells)	IL-2, IL-12, IFN-γ, and TNFα
CD4^+^ (Humoral Response)	Antigen presenting cell and epithelial signaling (IL-33, IL-25, and TSLP)	STAT6, GATA3	Th 2 Immunity (Recruit/activation of eosinophils, basophils, mast cells, and B cells	IL-4, IL-5, IL-9, IL-10, IL-13, and IL-25
CD8+ (Cytotoxic T)	Antigen exposure	Tbet, EOMES, RUNX3	Intracellular pathogen defense, tumor surveillance	(a) TNF-α, IFN-γ secretion; (b) cytotoxic granule release; (c) direct cytotoxicity (Fas/FasL)
Th9	IL-4 and TGF-β	STAT6, PU.1	Anti-parasitic	IL-9
Th17	Antigen dependent and independent activation, IL-6 and IL-23	RORγt, STAT3		(IL-17A, IL-17F, IL-21, and IL-22)
Th25/Treg	TGF-β	FOXP3	Immunosuppressive; prevent autoimmunity	IL-10, TGFβ, IL-35
Tissue resident memory T (TRM)	Pathogen exposure, epithelial signaling (IL-25)	BCL6, Blimp-1	Immunological memory	IL-5, IL-17, and CCR7
Unconventional T (Innate-like, NKTs, IELs, MAITs, γδTs)	IL-33, IL-25, TSLP	PLZF, RORγt, Tbet		IFNγ (all); IL-4, IL-13 (MAIT); IL-17 (MAIT, γδT);
**References**: (304–310)				

*Table describes the milieu necessary to trigger phenotype; transcription factors involved in subset activation; biological function; and activation signature. Appropriate references are listed at the bottom of the table*.

Lymphocyte senescence, and the health associated effects it produces, is the product of direct (cellular dysfunction) and indirect (declining T cell selection machinery; exposure) mechanisms. Clinical and experimental evidence indicate that CD57 and KLRG-1 expression defines senescent conventional T cells (316, 318). Epigenetic analysis of aged senescent lymphocytes demonstrate progressive expansion of reactive effector cells (i.e., Th17) through DNA hypermethylation of transcriptional regulators essential to T cell responses (LGALS1, IFNG, CCL5, GZMH, CCR7, CD27, and CD248) and differentiation (SATB1, TCF7, BCL11B, and RUNX3) (319). These responses are complemented by locus specific chromatin remodeling favoring terminal differentiation of naïve T cells into effector cells (CD8 and Th17) (320). As a result, evaluation of Th17-to-Treg ratio in aging cohorts indicates progressive skewing toward effector Th17^+^ (high affinity for self-antigens) in lieu of suppressive Tregs (312, 321–323). Similarly, aging is associated with progressive loss in mucosal-associated invariant T cell and iNKT numbers and variant diversity due to functional decline in CD8 negative/double negative ratio in the thymus of aged cohorts (324–326). Further analysis also identifies time related shift in CD4^+^ MAIT, and within this subtype a decrease in interferon IFNγ/IL-4 ratio indicating Th1 to Th2 transition (327). Their importance should not be understated, as they represent up to 10% of circulating T cells and accumulate in large numbers in the bowel and airways and perform central role in epithelium protection from pathogens (328). Notably, clinical and experimental evidence suggested they play juxtaposing role in homeostasis (protective) and chronic kidney, bowel and non-alcoholic liver disease (profibrotic) (329–331). In the context of asthma, MAITs communicate with B cells and eosinophils to promote allergen-induced airway inflammation (332). The latter function is shared with iNKT cells, a population for which we have more detailed information. These effector cells have been shown to promote T cell dysfunction in aging murine cohorts through proliferative inhibition of splenic T cells (333). Their intrinsic (functional) senescence is still not well-understood. However, clinical and experimental evidence shows that cigarette smoke promotes their accumulation and activation in the lung, a response linked to the development of COPD (334, 335). Nevertheless, iNKT cell's role in recognizing and clearing senescent cells may unlock their potential in attenuating progression of already established age-related disorders (336).

Experimental modeling of environmental and occupational hazard exposure corroborates the notion that external stress accelerates T cell biological senescence. For instance, inhalation of vanadium fumes, used as an additive in the production of steel, and exposure to titanium oxide nanoparticles impacts “normal” T cell selection and differentiation by depleting thymic antigen presenting cells and extensive inflammation (337, 338). Chronic inflammation induced by particulate matter exposure triggers T cell senescence, with external stimuli or neighboring senescent cells producing an inflammatory microenvironment (SASP) that reprograms the metabolic machinery of CD8^+^ T cells by boosting mitochondrial biogenesis, and thus ROS production, distinctively defined as inflamm-aging (339). Cigarette smoke from human studies show an increase in inflammatory CD4+ Th17 lymphocytes at blood- and pulmonary level in smokers (340, 341).

At the crossroads between innate and adaptive immunity, innate lymphoid cells represent a rare and powerful population that can shape the behavior of surrounding cells during stressful conditions. Of the three subtypes described to date, ILC2 and ILC3 have been clearly implicated in lung injury, wound healing, and fibrogenesis. Their role is primarily linked to the production of mediators that promote extracellular matrix destruction and remodeling (IL-17, TGFβ, IL-5, IL-13) [extensively reviewed by (342, 343)]. Recent reports describe transcriptional and functional ILC2 senescence/exhaustion in the aging lung and brain, resulting in inability to restock the ILC2 pool in aged mice (344). Interestingly, these effects were, at least in part, alleviated by adoptive transfer of activated ILC2s (345). While identification of these tissue resident lymphocytes is progressing fairly rapidly thanks to single-cell techniques, analysis of their roles in specific disease states still at its infancy. Thus, further functional examination of these cells in the context of a senescent state (aging and fibrosis) represents a promising strategy to advance the field and perhaps therapy.

## Biological Signatures and Therapy

A few non-specific serum biomarker link disease progression and patient's survival to senescence (IL-6 and TNFα) or pulmonary fibrosis (SP-A, SP-D, KL-6, MMP-7), while epigenetic remodeling has gained considerable traction in early detection both conditions (346–348). Changes in global and site-specific DNA hypermethylation (gene silencing) patterns are well-described in the literature and are been considered therapeutically (349–351). Blood screening of elderly populations and individuals with interstitial lung abnormalities identified complex microRNA signatures linked to senescence, proliferation, cell survival, transcript processing, translation, and immune function (348, 352). While the overlap between PF and aging miRNA signatures is not currently available, a three-arm analysis examining young, aged, aged fibrotic lung could be very informative. At the transcriptional level, the Least Absolute Shrinkage and Selection Operator (LASSO) regression method was recently used to identify a signature spanning across age- and chemical-induced senescence. This machine learning approach using training datasets from chronic exposure to cigarette smoke and radiation, allowed to build a transcriptomic age model that accurately predicts chronological age in untreated mice and the deviations associated with certain exposures based on a 57-gene signature including Cyp1a1, Lcn2, MMPs, and immunoglobulins (87).

There is no panacea or elixir to counter the effects of age- or chemical-induced senescence. Nevertheless, recent evidence highlighted a number of bioactive nutrients, supplements and therapeutics (termed senolytics), that blunt oxidative damage and reprogram the cells' inflammatory, metabolic and death machinery (353). Two such chemicals are the macrolides azithromycin and rapamycin, shown to clear senescent fibroblasts and thus reduce SASP-related factors through autophagic modulation (354). The anti-diabetic drug metformin and the immune-suppressor rapamycin have shown significant affinity to modulate AMP-activated protein kinase (AMPK), thereby reducing cellular apoptosis, and extending cell longevity (355–357). Lastly, a long line of natural compounds (i.e., resveratrol, fisetin, piperlongumine, and quercetin) that activate Nrf2 (nuclear factor erythroid-derived 2-related factor 2) are shown to be cytoprotective while also inducing senescence or apoptosis in damaged and potentially precancerous cells (358). There is increasing evidence that these chemicals are effective beyond aging, with ample research focusing on their anti-cancer benefits. Examination in the context of fibrosis is still in its infancy, with clinical and in experimental data (bleomycin) supporting the feasibility of quercetin, as well as metformin, against fibrosis. This appears to be achieved via AMPK activation in myofibroblasts, enhanced mitochondrial biogenesis and regulation of apoptotic sensitivity, which aids reversal of SASP and collagen deposition (359–362).

A second potentially groundbreaking approach to reverse pulmonary fibrosis and aging induced senescence, is represented by allogeneic injections of mesenchymal stem cell/multipotent stromal cell/marrow stromal cell (MSCs). A number of experimental and clinical evidence reveals promising results in chronic pulmonary disease, fibrosis and age-related frailty (363–365). These cells can be obtained *in vitro* through expansion of adherent bone marrow mononuclear cells and may function as a trophic source to support immune-senescent inflammatory cells (366).

## Conclusions and Future Perspectives

This review exposes the wealth of evidence that pertains to aging, environmental exposure, and fibrosis. While demonstrating some degree of mechanistic redundancy across the spectrum of senescence, it also highlights a number of knowledge gaps that need to be addressed to impact human health (i.e., therapeutics). Based on this foundation, the next cycle of research questions should test: (1) whether senescence of the remodeled/fibrotic lung occurs faster and through the same mechanisms as an architecturally pristine one? (2) Since it is understood that age-related dysfunction lowers the threshold necessary to trigger an irreversible senescent phenotype, can we model and accurately predict such levels? (3a) Can we identify mutual factors involved in senescence across the spectrum of chemical exposure? (3b) Can we identify shared and exclusive factors that drive senescence across the lung disease spectrum? For instance, how does PF senescence compare to that observed in COPD, emphysema, asthma? (4) Can we modulate/reprogram the behavior and communication of specific cell types, and thus amplify an anti-senescent signal? (5) Can senolytics modulate seemingly irreversible changes in the fibrotic (and aging) lung?

As we look beyond the next decade, it is absolutely necessary that we boost our effort to define sex and hormonal differences as they relate to lung function, senescence, response to environmental toxicants and fibrosis. In the context of immune cell function, it is shown that females elicit an estrogen driven humoral response (Th2 like), while testosterone supports Th1 immunity (367). Peripheral blood analysis shows that aging males exhibit marked epigenomic alteration linked to naïve T and B cell decline and increased monocyte cytotoxicity (368). How these hormones, or their imbalance during menopause and andropause, support or protects the lung from exogenous stressors and disease is not well-understood. This is likely the most important puzzle piece to understand clinical datasets. Yet, there is a canyon-sized knowledge gap in front of us.

Although we are a long way away from getting all the answers, it is comforting to see an increasingly collaborative scientific community and frequent technological advancements that help us comprehensively study cell biology. The successes of the next decade of research lies in good hands.

## Author Contributions

AV prepared and edited the manuscript.

## Conflict of Interest

The author declares that the research was conducted in the absence of any commercial or financial relationships that could be construed as a potential conflict of interest.

## References

[1] Lowery EM Brubaker AL Kuhlmann E Kovacs EJ The aging lung. Clin Interv Aging. (2013) 8:1489–96. 10.2147/CIA.S51152 24235821 PMC3825547

[2] Meiners S Eickelberg O Königshoff M. Hallmarks of the ageing lung. Eur Respir J. (2015) 45:807. 10.1183/09031936.00186914 25657021

[3] Sgalla G Iovene B Calvello M Ori M Varone F Richeldi L. Idiopathic pulmonary fibrosis: pathogenesis and management. Respir Res. (2018) 19:32–32. 10.1186/s12931-018-0730-2 29471816 PMC5824456

[4] Raghu G Chen SY Yeh WS Maroni B Li Q Lee YC. Idiopathic pulmonary fibrosis in US Medicare beneficiaries aged 65 years and older: incidence, prevalence, and survival, 2001-11. Lancet Respir Med. (2014) 2:566–72. 10.1016/S2213-2600(14)70101-8 24875841

[5] Barratt SL Creamer A Hayton C Chaudhuri N. Idiopathic pulmonary fibrosis (IPF): an overview. J Clin Med. (2018) 7:201. 10.3390/jcm7080201 30082599 PMC6111543

[6] Nalysnyk L Cid-Ruzafa J Rotella P Esser D. Incidence and prevalence of idiopathic pulmonary fibrosis: review of the literature. Eur Respir Rev. (2012) 21:355–61. 10.1183/09059180.00002512 23204124 PMC9487229

[7] Hutchinson JP Mckeever TM Fogarty AW Navaratnam V Hubbard RB. Increasing global mortality from idiopathic pulmonary fibrosis in the twenty-first century. Ann Am Thorac Soc. (2014) 11:1176–85. 10.1513/AnnalsATS.201404-145OC 25165873

[8] Sauleda J Núñez B Sala E Soriano JB. Idiopathic pulmonary fibrosis: epidemiology, natural history, phenotypes. Med Sci. (2018) 6:110. 10.3390/medsci6040110 30501130 PMC6313500

[9] Bowdish DME. The aging lung: is lung health good health for older adults? Chest. (2019) 155:391–400. 10.1016/j.chest.2018.09.003 30253136

[10] Cho SJ Stout-Delgado HW Aging and lung disease. Annu Rev Physiol. (2020) 82:433–59. 10.1146/annurev-physiol-021119-034610 31730381 PMC7998901

[11] Sharma G Goodwin J. Effect of aging on respiratory system physiology and immunology. Clin Interv Aging. (2006) 1:253–60. 10.2147/ciia.2006.1.3.253 18046878 PMC2695176

[12] Thannickal VJ Murthy M Balch WE Chandel NS Meiners S Eickelberg O. Blue journal conference. Aging and susceptibility to lung disease. Am J Respir Crit Care Med. (2015) 191:261–9. 10.1164/rccm.201410-1876PP 25590812 PMC4351581

[13] Gulati S Thannickal VJ. The aging lung and idiopathic pulmonary fibrosis. Am J Med Sci. (2019) 357:384–9. 10.1016/j.amjms.2019.02.008 31010465

[14] Guzik TJ Mohiddin SA Dimarco A Patel V Savvatis K Marelli-Berg FM. COVID-19 and the cardiovascular system: implications for risk assessment, diagnosis, and treatment options. Cardiovasc Res. (2020) 116:1666–87. 10.1093/cvr/cvaa106 32352535 PMC7197627

[15] Liu K Chen Y Lin R Han K. Clinical features of COVID-19 in elderly patients: a comparison with young and middle-aged patients. J Infect. (2020) 80:e14–8. 10.1016/j.jinf.2020.03.005 32171866 PMC7102640

[16] Marhl M Grubelnik V Magdič M Markovič R. Diabetes and metabolic syndrome as risk factors for COVID-19. Diabetes Metab Syndr. (2020) 14:671–7. 10.1016/j.dsx.2020.05.013 32438331 PMC7205616

[17] Hanahan D Weinberg RA. Hallmarks of cancer: the next generation. Cell. (2011) 144:646–74. 10.1016/j.cell.2011.02.013 21376230

[18] Glass D Viñuela A Davies MN Ramasamy A Parts L Knowles D. Gene expression changes with age in skin, adipose tissue, blood and brain. Genome Biol. (2013) 14:R75. 10.1186/gb-2013-14-7-r75 23889843 PMC4054017

[19] López-Otín C Blasco MA Partridge L Serrano M Kroemer G. The hallmarks of aging. Cell. (2013) 153:1194–217. 10.1016/j.cell.2013.05.039 23746838 PMC3836174

[20] Weidner CI Lin Q Koch CM Eisele L Beier F Ziegler P. Aging of blood can be tracked by DNA methylation changes at just three CpG sites. Genome Biol. (2014) 15:R24. 10.1186/gb-2014-15-2-r24 24490752 PMC4053864

[21] Yang J Huang T Petralia F Long Q Zhang B Argmann C. Synchronized age-related gene expression changes across multiple tissues in human and the link to complex diseases. Sci Rep. (2015) 5:15145. 10.1038/srep15145 26477495 PMC4609956

[22] Horvath S Raj K. DNA methylation-based biomarkers and the epigenetic clock theory of ageing. Nat Rev Genet. (2018) 19:371–84. 10.1038/s41576-018-0004-3 29643443

[23] Lu AT Xue L Salfati EL Chen BH Ferrucci L Levy D. GWAS of epigenetic aging rates in blood reveals a critical role for TERT. Nat Commun. (2018) 9:387. 10.1038/s41467-017-02697-5 29374233 PMC5786029

[24] Fulda S Gorman AM Hori O Samali A Cellular stress responses: cell survival and cell death. Int J Cell Biol. (2010) 2010:214074 10.1155/2010/214074 20182529 PMC2825543

[25] Zhang X Lin S Funk WE Hou L. Environmental and occupational exposure to chemicals and telomere length in human studies. Occup Environ Med. (2013) 70:743–9. 10.1136/oemed-2012-101350 23775864

[26] Martens DS Cox B Janssen BG Clemente DBP Gasparrini A Vanpoucke C. Prenatal air pollution and newborns' predisposition to accelerated biological aging. JAMA Pediatr. (2017) 171:1160–7. 10.1001/jamapediatrics.2017.3024 29049509 PMC6233867

[27] Correia-Melo C Marques FD Anderson R Hewitt G Hewitt R Cole J. Mitochondria are required for pro-ageing features of the senescent phenotype. EMBO J. (2016) 35:724–42. 10.15252/embj.201592862 26848154 PMC4818766

[28] Aivazidis S Anderson CC Roede JR. Toxicant-mediated redox control of proteostasis in neurodegeneration. Curr Opin Toxicol. (2019) 13:22–34. 10.1016/j.cotox.2018.12.007 31602419 PMC6785977

[29] Gorgoulis V Adams PD Alimonti A Bennett DC Bischof O Bishop C. Cellular senescence: defining a path forward. Cell. (2019) 179:813–27. 10.1016/j.cell.2019.10.005 31675495

[30] Gowans GJ Hardie DG. AMPK: a cellular energy sensor primarily regulated by AMP. Biochem Soc Trans. (2014) 42:71–5. 10.1042/BST20130244 24450630 PMC5703408

[31] Korolchuk VI Miwa S Carroll B Von Zglinicki T. Mitochondria in cell senescence: is mitophagy the weakest link? EBioMedicine. (2017) 21:7–13. 10.1016/j.ebiom.2017.03.020 28330601 PMC5514379

[32] Wu J Jin Z Yan LJ. Redox imbalance and mitochondrial abnormalities in the diabetic lung. Redox Biol. (2017) 11:51–9. 10.1016/j.redox.2016.11.003 27888691 PMC5124358

[33] Zhao H Dennery PA Yao H. Metabolic reprogramming in the pathogenesis of chronic lung diseases, including BPD, COPD, and pulmonary fibrosis. Am J Physiol Lung Cell Mol Physiol. (2018) 314:L544–54. 10.1152/ajplung.00521.2017 29351437 PMC5966782

[34] Fan J Krautkramer KA Feldman JL Denu JM Metabolic regulation of histone post-translational modifications. ACS Chem Biol. (2015) 10:95–108. 10.1021/cb500846u 25562692 PMC4407823

[35] Feron O. The many metabolic sources of acetyl-CoA to support histone acetylation and influence cancer progression. Ann Transl Med. (2019) 7(Suppl.):S277. 10.21037/atm.2019.11.140 32015996 PMC6976516

[36] Yamane K Mizuguchi T Cui B Zofall M Noma K-I Grewal SIS. Asf1/HIRA facilitate global histone deacetylation and associate with HP1 to promote nucleosome occupancy at heterochromatic loci. Mol Cell. (2011) 41:56–66. 10.1016/j.molcel.2010.12.009 21211723 PMC3035992

[37] Rai TS Cole JJ Nelson DM Dikovskaya D Faller WJ Vizioli MG. HIRA orchestrates a dynamic chromatin landscape in senescence and is required for suppression of neoplasia. Genes Dev. (2014) 28:2712–25. 10.1101/gad.247528.114 25512559 PMC4265675

[38] Acosta JC O'loghlen A Banito A Guijarro MV Augert A Raguz S. Chemokine signaling via the CXCR2 receptor reinforces senescence. Cell. (2008) 133:1006–18. 10.1016/j.cell.2008.03.038 18555777

[39] Bhaumik D Scott GK Schokrpur S Patil CK Orjalo AV Rodier F. MicroRNAs miR-146a/b negatively modulate the senescence-associated inflammatory mediators IL-6 and IL-8. Aging. (2009) 1:402–11. 10.18632/aging.100042 20148189 PMC2818025

[40] Coppé J-P Desprez P-Y Krtolica A Campisi J. The senescence-associated secretory phenotype: the dark side of tumor suppression. Annu Rev Pathol. (2010) 5:99–118. 10.1146/annurev-pathol-121808-102144 20078217 PMC4166495

[41] Campisi J D'adda Di Fagagna F. Cellular senescence: when bad things happen to good cells. Nat Rev Mol Cell Biol. (2007) 8:729–40. 10.1038/nrm2233 17667954

[42] Faner R Rojas M Macnee W Agustí A. Abnormal lung aging in chronic obstructive pulmonary disease and idiopathic pulmonary fibrosis. Am J Respir Crit Care Med. (2012) 186:306–13. 10.1164/rccm.201202-0282PP 22582162

[43] Chen R Zhang K Chen H Zhao X Wang J Li L. Telomerase deficiency causes alveolar stem cell senescence-associated low-grade inflammation in lungs. J Biol Chem. (2015) 290:30813–29. 10.1074/jbc.M115.681619 26518879 PMC4692211

[44] Ruwisch J Sehlmeyer K Roldan N Garcia-Alvarez B Perez-Gil J Weaver TE. Air space distension precedes spontaneous fibrotic remodeling and impaired cholesterol metabolism in the absence of surfactant protein C. Am J Respir Cell Mol Biol. (2020) 62:466–78. 10.1165/rcmb.2019-0358OC 31922895

[45] Selman M King TE Pardo A. Idiopathic pulmonary fibrosis: prevailing and evolving hypotheses about its pathogenesis and implications for therapy. Ann Intern Med. (2001) 134:136–51. 10.7326/0003-4819-134-2-200101160-00015 11177318

[46] Stevens PA Pettenazzo A Brasch F Mulugeta S Baritussio A Ochs M. Nonspecific interstitial pneumonia, alveolar proteinosis, and abnormal proprotein trafficking resulting from a spontaneous mutation in the surfactant protein C gene. Pediatr Res. (2005) 57:89–98. 10.1203/01.PDR.0000147567.02473.5A 15557112

[47] Diaz De Leon A Cronkhite JT Katzenstein A-LA Godwin JD Raghu G Glazer CS. Telomere lengths, pulmonary fibrosis and telomerase (TERT) mutations. PLoS ONE. (2010) 5:e10680. 10.1371/journal.pone.0010680 20502709 PMC2873288

[48] Young LR Gulleman PM Short CW Tanjore H Sherrill T Qi A. Epithelial-macrophage interactions determine pulmonary fibrosis susceptibility in Hermansky-Pudlak syndrome. JCI Insight. (2016) 1:e88947. 10.1172/jci.insight.88947 27777976 PMC5070955

[49] Nureki SI Tomer Y Venosa A Katzen J Russo SJ Jamil S. Expression of mutant Sftpc in murine alveolar epithelia drives spontaneous lung fibrosis. J Clin Invest. (2018) 128:4008–24. 10.1172/JCI99287 29920187 PMC6118576

[50] Bilgili H Białas AJ Górski P Piotrowski WJ. Telomere abnormalities in the pathobiology of idiopathic pulmonary fibrosis. J Clin Med. (2019) 8:1232. 10.3390/jcm8081232 31426295 PMC6723768

[51] Liu T Gonzalez De Los Santos F Zhao Y Wu Z Rinke AE Kim KK. Telomerase reverse transcriptase ameliorates lung fibrosis by protecting alveolar epithelial cells against senescence. J Biol Chem. (2019) 294:8861–71. 10.1074/jbc.RA118.006615 31000627 PMC6552422

[52] Uejima Y Fukuchi Y Nagase T Tabata R Orimo H. A new murine model of aging lung: the senescence accelerated mouse (SAM)-P. Mech Ageing Dev. (1991) 61:223–36. 10.1016/0047-6374(91)90057-7 1795564

[53] Sime PJ Xing Z Graham FL Csaky KG Gauldie J. Adenovector-mediated gene transfer of active transforming growth factor-beta1 induces prolonged severe fibrosis in rat lung. J Clin Invest. (1997) 100:768–76. 10.1172/JCI119590 9259574 PMC508247

[54] Lee CG Homer RJ Zhu Z Lanone S Wang X Koteliansky V. Interleukin-13 induces tissue fibrosis by selectively stimulating and activating transforming growth factor beta(1). J Exp Med. (2001) 194:809–21. 10.1084/jem.194.6.809 11560996 PMC2195954

[55] Young LR Gulleman PM Bridges JP Weaver TE Deutsch GH Blackwell TS. The alveolar epithelium determines susceptibility to lung fibrosis in Hermansky-Pudlak syndrome. Am J Respir Crit Care Med. (2012) 186:1014–24. 10.1164/rccm.201207-1206OC 23043085 PMC3530211

[56] Alder JK Barkauskas CE Limjunyawong N Stanley SE Kembou F Tuder RM. Telomere dysfunction causes alveolar stem cell failure. Proc Natl Acad Sci USA. (2015) 112:5099–104. 10.1073/pnas.1504780112 25840590 PMC4413294

[57] Venosa A Katzen J Tomer Y Kopp M Jamil S Russo SJ. Epithelial expression of an interstitial lung disease–associated mutation in surfactant protein-C modulates recruitment and activation of key myeloid cell populations in mice. J Immunol. (2019) 202:2760–71. 10.4049/jimmunol.1900039 30910861 PMC6478557

[58] Hay J Shahzeidi S Laurent G. Mechanisms of bleomycin-induced lung damage. Arch Toxicol. (1991) 65:81–94. 10.1007/BF02034932 1711838

[59] Bonner JC. Nanoparticles as a potential cause of pleural and interstitial lung disease. Proc Am Thorac Soc. (2010) 7:138–41. 10.1513/pats.200907-061RM 20427587 PMC3266021

[60] Moore BB Lawson WE Oury TD Sisson TH Raghavendran K Hogaboam CM. Animal models of fibrotic lung disease. Am J Respir Cell Mol Biol. (2013) 49:167–79. 10.1165/rcmb.2013-0094TR 23526222 PMC3824038

[61] Lu X Zhu T Chen C Liu Y Right or left: the role of nanoparticles in pulmonary diseases. Int J Mol Sci. (2014) 15:17577–600. 10.3390/ijms151017577 25268624 PMC4227179

[62] Beach TA Groves AM Johnston CJ Williams JP Finkelstein JN. Recurrent DNA damage is associated with persistent injury in progressive radiation-induced pulmonary fibrosis. Int J Radiat Biol. (2018) 94:1104–15. 10.1080/09553002.2018.1516907 30238842 PMC6309234

[63] Mumby S Chung KF Adcock IM. Transcriptional effects of ozone and impact on airway inflammation. Front Immunol. (2019) 10:1610. 10.3389/fimmu.2019.01610 31354743 PMC6635463

[64] Reid CE Considine EM Watson GL Telesca D Pfister GG Jerrett M. Associations between respiratory health and ozone and fine particulate matter during a wildfire event. Environ Int. (2019) 129:291–8. 10.1016/j.envint.2019.04.033 31146163

[65] Tovar A Smith GJ Thomas JM Crouse WL Harkema JR Kelada SNP. Transcriptional profiling of the murine airway response to acute ozone exposure. Toxicol Sci. (2019) 173:114–30. 10.1093/toxsci/kfz219 31626304 PMC6944221

[66] Parrinello S Samper E Krtolica A Goldstein J Melov S Campisi J. Oxygen sensitivity severely limits the replicative lifespan of murine fibroblasts. Nat Cell Biol. (2003) 5:741–7. 10.1038/ncb1024 12855956 PMC4940195

[67] Kohman RA Crowell B Kusnecov AW. Differential sensitivity to endotoxin exposure in young and middle-age mice. Brain Behav Immun. (2010) 24:486–92. 10.1016/j.bbi.2009.12.004 20025957 PMC2826540

[68] Seol M-A Jung U Eom HS Kim S-H Park H-R Jo S-K. Prolonged expression of senescence markers in mice exposed to gamma-irradiation. J Vet Sci. (2012) 13:331–8. 10.4142/jvs.2012.13.4.331 23271173 PMC3539117

[69] Hamsanathan S Alder JK Sellares J Rojas M Gurkar AU Mora AL. Cellular senescence: the trojan horse in chronic lung diseases. Am J Respir Cell Mol Biol. (2019) 61:21–30. 10.1165/rcmb.2018-0410TR 30965013 PMC6604222

[70] Islam JY Keller RL Aschner JL Hartert TV Moore PE. Understanding the short- and long-term respiratory outcomes of prematurity and bronchopulmonary dysplasia. Am J Respir Crit Care Med. (2015) 192:134–56. 10.1164/rccm.201412-2142PP 26038806 PMC4532824

[71] Savran O Ulrik CS. Early life insults as determinants of chronic obstructive pulmonary disease in adult life. Int J Chron Obstruct Pulmon Dis. (2018) 13:683–93. 10.2147/COPD.S153555 29520136 PMC5834168

[72] Apostol AC Jensen KDC Beaudin AE. Training the fetal immune system through maternal inflammation-a layered hygiene hypothesis. Front Immunol. (2020) 11:123. 10.3389/fimmu.2020.00123 32117273 PMC7026678

[73] van Bever HPS. Determinants in early life for asthma development. Allergy Asthma Clin Immunol. (2009) 5:6–6. 10.1186/1710-1492-5-6 20016777 PMC2794849

[74] Sookoian S Gianotti TF Burgueño AL Pirola CJ. Fetal metabolic programming and epigenetic modifications: a systems biology approach. Pediatr Res. (2013) 73:531–42. 10.1038/pr.2013.2 23314294

[75] Pascoe CD. Unravelling the impact of early life exposures on lung structure and function in the developmental origins of asthma. Respirology. (2017) 22:1241–2. 10.1111/resp.13098 28590023

[76] Deodati A Inzaghi E Cianfarani S. Epigenetics and *in utero* acquired predisposition to metabolic disease. Front Genet. (2020) 10:1270. 10.3389/fgene.2019.01270 32082357 PMC7000755

[77] Parikh P Wicher S Khandalavala K Pabelick CM Britt RD Jr Prakash YS. Cellular senescence in the lung across the age spectrum. Am J Physiol Lung Cell Mol Physiol. (2019) 316:L826–42. 10.1152/ajplung.00424.2018 30785345 PMC6589594

[78] Yuan L Du X Tang S Wu S Wang L Xiang Y. ITGB4 deficiency induces senescence of airway epithelial cells through p53 activation. FEBS J. (2019) 286:1191–203. 10.1111/febs.14749 30636108

[79] Connor AJ Laskin JD Laskin DL. Ozone-induced lung injury and sterile inflammation. Role of toll-like receptor 4. Exp Mol Pathol. (2012) 92:229–35. 10.1016/j.yexmp.2012.01.004 22300504 PMC3507381

[80] Groves AM Gow AJ Massa CB Hall L Laskin JD Laskin DL. Age-related increases in ozone-induced injury and altered pulmonary mechanics in mice with progressive lung inflammation. Am J Physiol Lung Cell Mol Physiol. (2013) 305:L555–68. 10.1152/ajplung.00027.2013 23997172 PMC3798776

[81] Gabehart K Correll KA Loader JE White CW Dakhama A. The lung response to ozone is determined by age and is partially dependent on toll-like receptor 4. Respir Res. (2015) 16:117. 10.1186/s12931-015-0279-2 26410792 PMC4583721

[82] Wiegman CH Michaeloudes C Haji G Narang P Clarke CJ Russell KE. Oxidative stress-induced mitochondrial dysfunction drives inflammation and airway smooth muscle remodeling in patients with chronic obstructive pulmonary disease. J Allergy Clin Immunol. (2015) 136:769–80. 10.1016/j.jaci.2015.01.046 25828268 PMC4559140

[83] Dong J Ma Q. Type 2 immune mechanisms in carbon nanotube-induced lung fibrosis. Front Immunol. (2018) 9:1120. 10.3389/fimmu.2018.01120 29872441 PMC5972321

[84] Galiè M Covi V Tabaracci G Malatesta M. The role of Nrf2 in the antioxidant cellular response to medical ozone exposure. Int J Mol Sci. (2019) 20:4009. 10.3390/ijms20164009 31426459 PMC6720777

[85] Shoeb M Mustafa GM Joseph P Umbright C Kodali V Roach KA. Initiation of pulmonary fibrosis after silica inhalation in rats is linked with dysfunctional shelterin complex and DNA damage response. Sci Rep. (2019) 9:471. 10.1038/s41598-018-36712-6 30679488 PMC6346028

[86] Zhang N Liu K Wang K Zhou C Wang H Che S. Dust induces lung fibrosis through dysregulated DNA methylation. Environ Toxicol. (2019) 34:728–41. 10.1002/tox.22739 30815999

[87] Choukrallah M-A Hoeng J Peitsch MC Martin F. Lung transcriptomic clock predicts premature aging in cigarette smoke-exposed mice. BMC Genomics. (2020) 21:291. 10.1186/s12864-020-6712-z 32272900 PMC7147004

[88] Jeyapalan JC Ferreira M Sedivy JM Herbig U. Accumulation of senescent cells in mitotic tissue of aging primates. Mech Ageing Dev. (2007) 128:36–44. 10.1016/j.mad.2006.11.008 17116315 PMC3654105

[89] Panda A Arjona A Sapey E Bai F Fikrig E Montgomery RR. Human innate immunosenescence: causes and consequences for immunity in old age. Trends Immunol. (2009) 30:325–33. 10.1016/j.it.2009.05.004 19541535 PMC4067971

[90] Signer RA Morrison SJ. Mechanisms that regulate stem cell aging and life span. Cell Stem Cell. (2013) 12:152–65. 10.1016/j.stem.2013.01.001 23395443 PMC3641677

[91] Hollingsworth JW Kleeberger SR Foster WM. Ozone and pulmonary innate immunity. Proc Am Thorac Soc. (2007) 4:240–6. 10.1513/pats.200701-023AW 17607006 PMC2647625

[92] Yang Q Ge MQ Kokalari B Redai IG Wang X Kemeny DM. Group 2 innate lymphoid cells mediate ozone-induced airway inflammation and hyperresponsiveness in mice. J Allergy Clin Immunol. (2016) 137:571–8. 10.1016/j.jaci.2015.06.037 26282284 PMC4747855

[93] Francis M Groves AM Sun R Cervelli JA Choi H Laskin JD. Editor's highlight: CCR2 Regulates inflammatory cell accumulation in the lung and tissue injury following ozone exposure. Toxicol Sci. (2017) 155:474–84. 10.1093/toxsci/kfw226 27837169 PMC5291213

[94] Kumagai K Lewandowski RP Jackson-Humbles DN Buglak N Li N White K. Innate Lymphoid cells mediate pulmonary eosinophilic inflammation, airway mucous cell metaplasia, and type 2 immunity in mice exposed to ozone. Toxicol Pathol. (2017) 45:692–704. 10.1177/0192623317728135 28891433

[95] Michaudel C Fauconnier L Julé Y Ryffel B. Functional and morphological differences of the lung upon acute and chronic ozone exposure in mice. Sci Rep. (2018) 8:10611. 10.1038/s41598-018-28261-9 30006538 PMC6045627

[96] Massa CB Groves AM Jaggernauth SU Laskin DL Gow AJ. Histologic and biochemical alterations predict pulmonary mechanical dysfunction in aging mice with chronic lung inflammation. PLoS Comput Biol. (2017) 13:e1005570. 10.1371/journal.pcbi.1005570 28837561 PMC5570219

[97] Franks TJ Colby TV Travis WD Tuder RM Reynolds HY Brody AR. Resident cellular components of the human lung: current knowledge and goals for research on cell phenotyping and function. Proc Am Thorac Soc. (2008) 5:763–6. 10.1513/pats.200803-025HR 18757314

[98] Mulugeta S Nureki S Beers MF. Lost after translation: insights from pulmonary surfactant for understanding the role of alveolar epithelial dysfunction and cellular quality control in fibrotic lung disease. Am J Physiol Lung Cell Mol Physiol. (2015) 309:L507–525. 10.1152/ajplung.00139.2015 26186947 PMC4572416

[99] Beers MF Knudsen L Tomer Y Maronn J Zhao M Ochs M. Aberrant lung remodeling in a mouse model of surfactant dysregulation induced by modulation of the Abca3 gene. Ann Anat. (2017) 210:135–46. 10.1016/j.aanat.2016.11.015 28034695 PMC5579840

[100] Aran D Looney AP Liu L Wu E Fong V Hsu A. Reference-based analysis of lung single-cell sequencing reveals a transitional profibrotic macrophage. Nat Immunol. (2019) 20:163–72. 10.1038/s41590-018-0276-y 30643263 PMC6340744

[101] Trethewey SP Walters GI. The role of occupational and environmental exposures in the pathogenesis of idiopathic pulmonary fibrosis: a narrative literature review. Medicina. (2018) 54:108. 10.3390/medicina54060108 30544758 PMC6306764

[102] Winterbottom CJ Shah RJ Patterson KC Kreider ME Panettieri RA Jr Rivera-Lebron B. Exposure to ambient particulate matter is associated with accelerated functional decline in idiopathic pulmonary fibrosis. Chest. (2018) 153:1221–8. 10.1016/j.chest.2017.07.034 28802694 PMC6026290

[103] Cooper DM Loxham M. Particulate matter and the airway epithelium: the special case of the underground? Eur Respir Rev. (2019) 28:190066. 10.1183/16000617.0066-2019 31554704 PMC9488653

[104] Sun B Shi Y Li Y Jiang J Liang S Duan J. Short-term PM(2.5) exposure induces sustained pulmonary fibrosis development during post-exposure period in rats. J Hazard Mater. (2020) 385:121566. 10.1016/j.jhazmat.2019.121566 31761645

[105] Willis BC Dubois RM Borok Z. Epithelial origin of myofibroblasts during fibrosis in the lung. Proc Am Thorac Soc. (2006) 3:377–82. 10.1513/pats.200601-004TK 16738204 PMC2658689

[106] Jin X Su R Li R Cheng L Li Z. Crucial role of pro-inflammatory cytokines from respiratory tract upon PM(2.5) exposure in causing the BMSCs differentiation in cells and animals. Oncotarget. (2017) 9:1745–59. 10.18632/oncotarget.23158 29416728 PMC5788596

[107] Reynolds WJ Hanson PS Critchley A Griffiths B Chavan B Birch-Machin MA. Exposing human primary dermal fibroblasts to particulate matter induces changes associated with skin aging. FASEB J. (2020) 34:14725–35. 10.1096/fj.202001357R 32915475

[108] Barlow PG Brown DM Donaldson K Maccallum J Stone V. Reduced alveolar macrophage migration induced by acute ambient particle (PM10) exposure. Cell Biol Toxicol. (2007) 24:243. 10.1007/s10565-007-9033-y 17846904

[109] Deiuliis JA Kampfrath T Zhong J Oghumu S Maiseyeu A Chen LC. Pulmonary T cell activation in response to chronic particulate air pollution. Am J Physiol Lung Cell Mol Physiol. (2012) 302:L399–409. 10.1152/ajplung.00261.2011 22160305 PMC3289266

[110] Wu J-Z Ge D-D Zhou L-F Hou L-Y Zhou Y Li Q-Y. Effects of particulate matter on allergic respiratory diseases. Chronic Dis Transl Med. (2018) 4:95–102. 10.1016/j.cdtm.2018.04.001 29988900 PMC6034084

[111] Estrella B Naumova EN Cepeda M Voortman T Katsikis PD Drexhage HA. Effects of air pollution on lung innate lymphoid cells: review of *in vitro* and *in vivo* experimental studies. Int J Environ Res Public Health. (2019) 16:2347. 10.3390/ijerph16132347 31269777 PMC6650824

[112] Chen LC Hwang J-S. Effects of subchronic exposures to concentrated ambient particles (CAPs) in mice: IV. Characterization of acute and chronic effects of ambient air fine particulate matter exposures on heart-rate variability. Inhal Toxicol. (2005) 17:209–16. 10.1080/08958370590912789 15804938

[113] Vermylen J Nemmar A Nemery B Hoylaerts MF. Ambient air pollution and acute myocardial infarction. J Thromb Haemost. (2005) 3:1955–61. 10.1111/j.1538-7836.2005.01471.x 16102102

[114] Zhao R Guo Z Zhang R Deng C Xu J Dong W. Nasal epithelial barrier disruption by particulate matter ≤ 2.5 μm via tight junction protein degradation. J Appl Toxicol. (2018) 38:678–87. 10.1002/jat.3573 29235125

[115] Xian M Ma S Wang K Lou H Wang Y Zhang L. (2020). Particulate matter 2.5 causes deficiency in barrier integrity in human nasal epithelial cells. Allergy Asthma Immunol Res. 12:56–71. 10.4168/aair.2020.12.1.56 31743964 PMC6875480

[116] Veranth JM Moss TA Chow JC Labban R Nichols WK Walton JC. Correlation of *in vitro* cytokine responses with the chemical composition of soil-derived particulate matter. Environ Health Perspect. (2006) 114:341–9. 10.1289/ehp.8360 16507455 PMC1392226

[117] Rosas Pérez I Serrano J Alfaro-Moreno E Baumgardner D García-Cuellar C Martín Del Campo JM. Relations between PM10 composition and cell toxicity: a multivariate and graphical approach. Chemosphere. (2007) 67:1218–28. 10.1016/j.chemosphere.2006.10.078 17188738

[118] Nemmar A Holme JA Rosas I Schwarze PE Alfaro-Moreno E. Recent advances in particulate matter and nanoparticle toxicology: a review of the *in vivo* and *in vitro* studies. Biomed Res Int. (2013) 2013:279371. 10.1155/2013/279371 23865044 PMC3705851

[119] Fehrenbach H. Alveolar epithelial type II cell: defender of the alveolus revisited. Respir Res. (2001) 2:33–46. 10.1186/rr36 11686863 PMC59567

[120] Kalluri R Weinberg RA. The basics of epithelial-mesenchymal transition. J Clin Invest. (2009) 119:1420–8. 10.1172/JCI39104 19487818 PMC2689101

[121] Barkauskas CE Cronce MJ Rackley CR Bowie EJ Keene DR Stripp BR. Type 2 alveolar cells are stem cells in adult lung. J Clin Invest. (2013) 123:3025–36. 10.1172/JCI68782 23921127 PMC3696553

[122] Panduri V Liu G Surapureddi S Kondapalli J Soberanes S de Souza-Pinto NC. Role of mitochondrial hOGG1 and aconitase in oxidant-induced lung epithelial cell apoptosis. Free Radic Biol Med. (2009) 47:750–9. 10.1016/j.freeradbiomed.2009.06.010 19524665 PMC4331123

[123] Walski M Pokorski M Antosiewicz J Rekawek A Frontczak-Baniewicz M Jernajczyk U. Pulmonary surfactant: ultrastructural features and putative mechanisms of aging. J Physiol Pharmacol. (2009) 60(Suppl. 5):121–5. 20134052

[124] Whitsett JA Wert SE Weaver TE. Alveolar surfactant homeostasis and the pathogenesis of pulmonary disease. Annu Rev Med. (2010) 61:105–19. 10.1146/annurev.med.60.041807.123500 19824815 PMC4127631

[125] Duerr J Leitz DHW Szczygiel M Dvornikov D Fraumann SG Kreutz C. Conditional deletion of Nedd4-2 in lung epithelial cells causes progressive pulmonary fibrosis in adult mice. Nat Commun. (2020) 11:2012. 10.1038/s41467-020-15743-6 32332792 PMC7181726

[126] Yazicioglu T Mühlfeld C Autilio C Huang CK Bär C Dittrich-Breiholz O. Aging impairs alveolar epithelial type II cell function in acute lung injury. Am J Physiol Lung Cell Mol Physiol. (2020) 319:L755–69. 10.1152/ajplung.00093.2020 32877222

[127] Tsakiri KD Cronkhite JT Kuan PJ Xing C Raghu G Weissler JC. Adult-onset pulmonary fibrosis caused by mutations in telomerase. Proc Natl Acad Sci USA. (2007) 104:7552–7. 10.1073/pnas.0701009104 17460043 PMC1855917

[128] Haghverdi L Büttner M Wolf FA Buettner F Theis FJ. Diffusion pseudotime robustly reconstructs lineage branching. Nat Methods. (2016) 13:845–8. 10.1038/nmeth.3971 27571553

[129] Strunz M Simon LM Ansari M Kathiriya JJ Angelidis I Mayr CH. Alveolar regeneration through a Krt8+ transitional stem cell state that persists in human lung fibrosis. Nat Commun. (2020) 11:3559. 10.1038/s41467-020-17358-3 32678092 PMC7366678

[130] Choi J Park JE Tsagkogeorga G Yanagita M Koo BK Han N. Inflammatory signals induce AT2 cell-derived damage-associated transient progenitors that mediate alveolar regeneration. Cell Stem Cell. (2020 27:366–82.e7. 10.1016/j.stem.2020.06.020 32750316 PMC7487779

[131] Kobayashi Y Tata A Konkimalla A Katsura H Lee RF Ou J. Persistence of a regeneration-associated, transitional alveolar epithelial cell state in pulmonary fibrosis. Nat Cell Biol. (2020) 22:934–46. 10.1101/855155 32661339 PMC7461628

[132] Adams T Schupp J Poli S Ayaub E Neumark N Ahangari F. Single cell RNA-seq reveals ectopic and aberrant lung resident cell populations in idiopathic pulmonary fibrosis. Sci Adv. (2020) 6:eaba1983. 10.1126/sciadv.aba1983 32832599 PMC7439502

[133] Chilosi M Doglioni C Murer B Poletti V. Epithelial stem cell exhaustion in the pathogenesis of idiopathic pulmonary fibrosis. Sarcoidosis Vasc Diffuse Lung Dis. (2010) 27:7–18. 21086900

[134] Angelidis I Simon LM Fernandez IE Strunz M Mayr CH Greiffo FR. An atlas of the aging lung mapped by single cell transcriptomics and deep tissue proteomics. Nat Commun. (2019) 10:963. 10.1038/s41467-019-08831-9 30814501 PMC6393476

[135] Sicard D Haak AJ Choi KM Craig AR Fredenburgh LE Tschumperlin DJ. Aging and anatomical variations in lung tissue stiffness. Am J Physiol Lung Cell Mol Physiol. (2018) 314:L946–55. 10.1152/ajplung.00415.2017 29469613 PMC6032071

[136] Hashimoto M Asai A Kawagishi H Mikawa R Iwashita Y Kanayama K. Elimination of p19(ARF)-expressing cells enhances pulmonary function in mice. JCI Insight. (2016) 1:e87732. 10.1172/jci.insight.87732 27699227 PMC5033852

[137] Tian Y Li H Qiu T Dai J Zhang Y Chen J. Loss of PTEN induces lung fibrosis via alveolar epithelial cell senescence depending on NF-κB activation. Aging Cell. (2019) 18:e12858. 10.1111/acel.12858 30548445 PMC6351835

[138] Rana T Jiang C Liu G Miyata T Antony V Thannickal VJ. PAI-1 regulation of TGF-β1-induced Alveolar type II cell senescence, SASP secretion, and SASP-mediated activation of alveolar macrophages. Am J Respir Cell Mol Biol. (2020) 62:319–30. 10.1165/rcmb.2019-0071OC 31513752 PMC7055702

[139] Johannson KA Vittinghoff E Morisset J Wolters PJ Noth EM Balmes JR. Air Pollution exposure is associated with lower lung function, but not changes in lung function, in patients with idiopathic pulmonary fibrosis. Chest. (2018) 154:119–25. 10.1016/j.chest.2018.01.015 29355549 PMC6045778

[140] Sesé L Nunes H Cottin V Sanyal S Didier M Carton Z. Role of atmospheric pollution on the natural history of idiopathic pulmonary fibrosis. Thorax. (2018) 73:145–50. 10.1136/thoraxjnl-2017-209967 28798214

[141] Pardo A Selman M. Lung fibroblasts, aging, and idiopathic pulmonary fibrosis. Ann Am Thorac Soc. (2016) 13(Suppl. 5):S417–21. 10.1513/AnnalsATS.201605-341AW 28005427

[142] Jang J-H Bruse S Huneidi S Schrader RM Monick MM Lin Y. Acrolein-exposed normal human lung fibroblasts *in vitro*: cellular senescence, enhanced telomere erosion, and degradation of Werner's syndrome protein. Environ Health Perspect. (2014) 122:955–62. 10.1289/ehp.1306911 24747221 PMC4154210

[143] Chen X Xu H Hou J Wang H Zheng Y Li H. Epithelial cell senescence induces pulmonary fibrosis through Nanog-mediated fibroblast activation. Aging. (2019) 12:242–59. 10.18632/aging.102613 31891567 PMC6977687

[144] Waters DW Blokland KEC Pathinayake PS Wei L Schuliga M Jaffar J. STAT3 regulates the onset of oxidant-induced senescence in lung fibroblasts. Am J Respir Cell Mol Biol. (2019) 61:61–73. 10.1165/rcmb.2018-0328OC 30608861

[145] Torres-González E Bueno M Tanaka A Krug LT Cheng DS Polosukhin VV. Role of endoplasmic reticulum stress in age-related susceptibility to lung fibrosis. Am J Respir Cell Mol Biol. (2012) 46:748–56. 10.1165/rcmb.2011-0224OC 22227563 PMC3380287

[146] Hecker L Logsdon NJ Kurundkar D Kurundkar A Bernard K Hock T. Reversal of persistent fibrosis in aging by targeting Nox4-Nrf2 redox imbalance. Sci Transl Med. (2014) 6:231ra47. 10.1126/scitranslmed.3008182 24718857 PMC4545252

[147] Sueblinvong V Neveu WA Neujahr DC Mills ST Rojas M Roman J. Aging promotes pro-fibrotic matrix production and increases fibrocyte recruitment during acute lung injury. Adv Biosci Biotechnol. (2014) 5:19–30. 10.4236/abb.2014.51004 24596659 PMC3939026

[148] Xu J Gonzalez ET Iyer SS Mac V Mora AL Sutliff RL. Use of senescence-accelerated mouse model in bleomycin-induced lung injury suggests that bone marrow–derived cells can alter the outcome of lung injury in aged mice. J Gerontol Ser A. (2009) 64A:731–9. 10.1093/gerona/glp040 19359440 PMC2844132

[149] Yanai H Shteinberg A Porat Z Budovsky A Braiman A Ziesche R. Cellular senescence-like features of lung fibroblasts derived from idiopathic pulmonary fibrosis patients. Aging. (2015) 7:664–72. 10.18632/aging.100807 26399448 PMC4600624

[150] Álvarez D Cárdenes N Sellarés J Bueno M Corey C Hanumanthu VS. IPF lung fibroblasts have a senescent phenotype. Am J Physiol Lung Cell Mol Physiol. (2017) 313:L1164–73. 10.1152/ajplung.00220.2017 28860144 PMC6148001

[151] Park S-Y Byun EJ Lee JD Kim S Kim HS. Air pollution, autophagy, and skin aging: impact of particulate matter (PM(10)) on human dermal fibroblasts. Int J Mol Sci. (2018) 19:2727. 10.3390/ijms19092727 30213068 PMC6163910

[152] Chan YL Wang B Chen H Ho KF Cao J Hai G. Pulmonary inflammation induced by low-dose particulate matter exposure in mice. Am J Physiol Lung Cell Mol Physiol. (2019) 317:L424–30. 10.1152/ajplung.00232.2019 31364371 PMC6766715

[153] Schafer MJ White TA Iijima K Haak AJ Ligresti G Atkinson EJ. Cellular senescence mediates fibrotic pulmonary disease. Nat Commun. (2017) 8:14532. 10.1038/ncomms14532 28230051 PMC5331226

[154] Keating A. Mesenchymal stromal cells. Curr Opin Hematol. (2006) 13:419–25. 10.1097/01.moh.0000245697.54887.6f 17053453 PMC3365862

[155] Badri L Murray S Liu LX Walker NM Flint A Wadhwa A. Mesenchymal stromal cells in bronchoalveolar lavage as predictors of bronchiolitis obliterans syndrome. Am J Respir Crit Care Med. (2011) 183:1062–70. 10.1164/rccm.201005-0742OC 21169468 PMC3086744

[156] Popova AP Bozyk PD Bentley JK Linn MJ Goldsmith AM Schumacher RE. Isolation of tracheal aspirate mesenchymal stromal cells predicts bronchopulmonary dysplasia. Pediatrics. (2010) 126:e1127–33. 10.1542/peds.2009-3445 20937656 PMC3887445

[157] Salam N Rane S Das R Faulkner M Gund R Kandpal U. T cell ageing: effects of age on development, survival and function. Indian J Med Res. (2013) 138:595–608. 24434315 PMC3928693

[158] Franceschi C Capri M Monti D Giunta S Olivieri F Sevini F. Inflammaging and anti-inflammaging: a systemic perspective on aging and longevity emerged from studies in humans. Mech Ageing Dev. (2007) 128:92–105. 10.1016/j.mad.2006.11.016 17116321

[159] Childs BG Baker DJ Wijshake T Conover CA Campisi J van Deursen JM. Senescent intimal foam cells are deleterious at all stages of atherosclerosis. Science. (2016) 354:472–7. 10.1126/science.aaf6659 27789842 PMC5112585

[160] Özcan S Alessio N Acar MB Mert E Omerli F Peluso G Unbiased analysis of senescence associated secretory phenotype (SASP) to identify common components following different genotoxic stresses. Aging. (2016) 8:1316–29. 10.18632/aging.100971 27288264 PMC4993333

[161] Hochane M Trichet V Pecqueur C Avril P Oliver L Denis J Low-dose pesticide mixture induces senescence in normal mesenchymal *stem cells* (MSC) and promotes tumorigenic phenotype in premalignant MSC. Stem Cells. (2017) 35:800–11. 10.1002/stem.2539 27860054

[162] Cárdenes N Álvarez D Sellarés J Peng Y Corey C Wecht S Senescence of bone marrow-derived mesenchymal stem cells from patients with idiopathic pulmonary fibrosis. Stem Cell Res Ther. (2018) 9:257 10.1186/s13287-018-0970-6 30257725 PMC6158816

[163] Linehan E Fitzgerald DC. Ageing and the immune system: focus on macrophages. Eur J Microbiol Immunol. (2015) 5:14–24. 10.1556/EuJMI-D-14-00035 25883791 PMC4397845

[164] Pinti M Appay V Campisi J Frasca D Fülöp T Sauce D. Aging of the immune system: focus on inflammation and vaccination. Eur J Immunol. (2016) 46:2286–301. 10.1002/eji.201546178 27595500 PMC5156481

[165] Crespo J Sun H Welling TH Tian Z Zou W. T cell anergy, exhaustion, senescence, and stemness in the tumor microenvironment. Curr Opin Immunol. (2013) 25:214–21. 10.1016/j.coi.2012.12.003 23298609 PMC3636159

[166] Wherry EJ Kurachi M. Molecular and cellular insights into T cell exhaustion. Nat Rev Immunol. (2015) 15:486–99. 10.1038/nri3862 26205583 PMC4889009

[167] Balint B Haas J Schwarz A Jarius S Fürwentsches A Engelhardt K. T-cell homeostasis in pediatric multiple sclerosis: old cells in young patients. Neurology. (2013) 81:784–92. 10.1212/WNL.0b013e3182a2ce0e 23911752

[168] Wiley CD Brumwell AN Davis SS Jackson JR Valdovinos A Calhoun C. Secretion of leukotrienes by senescent lung fibroblasts promotes pulmonary fibrosis. JCI Insight. (2019) 4:e130056. 10.1172/jci.insight.130056 31687975 PMC6975274

[169] Leuschner G Behr J. Acute exacerbation in interstitial lung disease. Front Med. (2017) 4:176. 10.3389/fmed.2017.00176 29109947 PMC5660065

[170] Mokra D Mikolka P Kosutova P Mokry J. Corticosteroids in acute lung injury: the dilemma continues. Int J Mol Sci. (2019) 20:4765. 10.3390/ijms20194765 31557974 PMC6801694

[171] Pang WW Price EA Sahoo D Beerman I Maloney WJ Rossi DJ. Human bone marrow hematopoietic stem cells are increased in frequency and myeloid-biased with age. Proc Natl Acad Sci USA. (2011) 108:20012–7. 10.1073/pnas.1116110108 22123971 PMC3250139

[172] Kovtonyuk LV Fritsch K Feng X Manz MG Takizawa H. Inflamm-aging of hematopoiesis, hematopoietic stem cells, and the bone marrow microenvironment. Front Immunol. (2016) 7:502. 10.3389/fimmu.2016.00502 27895645 PMC5107568

[173] Jones CV Ricardo SD. Macrophages and CSF-1: implications for development and beyond. Organogenesis. (2013) 9:249–60. 10.4161/org.25676 23974218 PMC3903694

[174] Iqbal AJ Mcneill E Kapellos TS Regan-Komito D Norman S Burd S Human CD68 promoter GFP transgenic mice allow analysis of monocyte to macrophage differentiation *in vivo*. Blood. (2014) 124:e33–44. 10.1182/blood-2014-04-568691 25030063 PMC4192756

[175] Cuda CM Misharin AV Khare S Saber R Tsai F Archer AM. Conditional deletion of caspase-8 in macrophages alters macrophage activation in a RIPK-dependent manner. Arthritis Res Ther. (2015) 17:291. 10.1186/s13075-015-0794-z 26471282 PMC4608154

[176] Borthwick LA Barron L Hart KM Vannella KM Thompson RW Oland S. Macrophages are critical to the maintenance of IL-13-dependent lung inflammation and fibrosis. Muc Immunol. (2016) 9:38–55. 10.1038/mi.2015.34 25921340 PMC4626445

[177] Yu X Buttgereit A Lelios I Utz SG Cansever D Becher B. The cytokine TGF-beta promotes the development and homeostasis of alveolar macrophages. Immunity. (2017) 47:903–12.e4. 10.1016/j.immuni.2017.10.007 29126797

[178] Gibbings SL Goyal R Desch AN Leach SM Prabagar M Atif SM. Transcriptome analysis highlights the conserved difference between embryonic and postnatal-derived alveolar macrophages. Blood. (2015) 126:1357–66. 10.1182/blood-2015-01-624809 26232173 PMC4566811

[179] Misharin AV Morales-Nebreda L Reyfman PA Cuda CM Walter JM Mcquattie-Pimentel AC. Monocyte-derived alveolar macrophages drive lung fibrosis and persist in the lung over the life span. J Exp Med. (2017) 214:2387–404. 10.1084/jem.20162152 28694385 PMC5551573

[180] Napier BA Monack DM. Creating a RAW264.7 CRISPR-Cas9 genome wide library. Bio Protoc. (2017) 7:e2320. 10.21769/BioProtoc.2320 28868328 PMC5580966

[181] Luo YL Xu CF Li HJ Cao ZT Liu J Wang JL. Macrophage-specific *in vivo* gene editing using cationic lipid-assisted polymeric nanoparticles. ACS Nano. (2018) 12:994–1005. 10.1021/acsnano.7b07874 29314827

[182] Mantovani A Sica A Sozzani S Allavena P Vecchi A Locati M. The chemokine system in diverse forms of macrophage activation and polarization. Trends Immunol. (2004) 25:677–86. 10.1016/j.it.2004.09.015 15530839

[183] Gleissner CA Shaked I Little KM Ley K. CXC chemokine ligand 4 induces a unique transcriptome in monocyte-derived macrophages. J Immunol. (2010) 184:4810–8. 10.4049/jimmunol.0901368 20335529 PMC3418140

[184] Gleissner CA. Macrophage phenotype modulation by CXCL4 in atherosclerosis. Front Physiol. (2012) 3:1. 10.3389/fphys.2012.00001 22275902 PMC3257836

[185] Ferrante CJ Pinhal-Enfield G Elson G Cronstein BN Hasko G Outram S. The adenosine-dependent angiogenic switch of macrophages to an M2-like phenotype is independent of interleukin-4 receptor alpha (IL-4Rα) signaling. Inflammation. (2013) 36:921–31. 10.1007/s10753-013-9621-3 23504259 PMC3710311

[186] Colin S Chinetti-Gbaguidi G Staels B. Macrophage phenotypes in atherosclerosis. Immunol Rev. (2014) 262:153–66. 10.1111/imr.12218 25319333

[187] Galvan-Pena S O'neill LA. Metabolic reprograming in macrophage polarization. Front Immunol. (2014) 5:420. 10.3389/fimmu.2014.00420 25228902 PMC4151090

[188] Martinez FO Gordon S. The M1 and M2 paradigm of macrophage activation: time for reassessment. F1000Prime Rep. (2014) 6:13. 10.12703/P6-13 24669294 PMC3944738

[189] Chinetti-Gbaguidi G Colin S Staels B. Macrophage subsets in atherosclerosis. Nat Rev Cardiol. (2015) 12:10–7. 10.1038/nrcardio.2014.173 25367649

[190] Chistiakov DA Bobryshev YV Orekhov AN. Changes in transcriptome of macrophages in atherosclerosis. J Cell Mol Med. (2015) 19:1163–73. 10.1111/jcmm.12591 25973901 PMC4459832

[191] Roszer T. Understanding the mysterious M2 macrophage through activation markers and effector mechanisms. Mediat Inflamm. (2015) 2015:816460. 10.1155/2015/816460 26089604 PMC4452191

[192] Mills EL Kelly B Logan A Costa ASH Varma M Bryant CE. Succinate dehydrogenase supports metabolic repurposing of mitochondria to drive inflammatory macrophages. Cell. (2016) 167:457–70.e413. 10.1016/j.cell.2016.08.064 27667687 PMC5863951

[193] Stienstra R Netea-Maier RT Riksen NP Joosten LAB Netea MG. Specific and complex reprogramming of cellular metabolism in myeloid cells during innate immune responses. Cell Metab. (2017) 26:142–56. 10.1016/j.cmet.2017.06.001 28683282

[194] Parisi L Gini E Baci D Tremolati M Fanuli M Bassani B Macrophage polarization in chronic inflammatory diseases: killers or builders? J Immunol Res. (2018) 2018:8917804 10.1155/2018/8917804 29507865 PMC5821995

[195] Ruytinx P Proost P van Damme J Struyf S. Chemokine-induced macrophage polarization in inflammatory conditions. Front Immunol. (2018) 9:1930. 10.3389/fimmu.2018.01930 30245686 PMC6137099

[196] Laskin DL Malaviya R Laskin JD. Role of macrophages in acute lung injury and chronic fibrosis induced by pulmonary toxicants. Toxicol Sci. (2019) 168:287–301. 10.1093/toxsci/kfy309 30590802 PMC6432864

[197] Yao Y Xu X-H Jin L. Macrophage polarization in physiological and pathological pregnancy. Front Immunol. (2019) 10:792. 10.3389/fimmu.2019.00792 31037072 PMC6476302

[198] Mahbub S Deburghgraeve CR Kovacs EJ. Advanced age impairs macrophage polarization. J Interferon Cytok Res. (2012) 32:18–26. 10.1089/jir.2011.0058 22175541 PMC3255514

[199] Cudejko C Wouters K Fuentes L Hannou SA Paquet C Bantubungi K. p16INK4a deficiency promotes IL-4-induced polarization and inhibits proinflammatory signaling in macrophages. Blood. (2011) 118:2556–66. 10.1182/blood-2010-10-313106 21636855 PMC3677739

[200] Mosser DM Edwards JP. Exploring the full spectrum of macrophage activation. Nat Rev Immunol. (2008) 8:958–69. 10.1038/nri2448 19029990 PMC2724991

[201] Morganti JM Riparip LK Rosi S. Call off the dog(ma): M1/M2 polarization is concurrent following traumatic brain injury. PLoS ONE. (2016) 11:e0148001. 10.1371/journal.pone.0148001 26808663 PMC4726527

[202] Misharin AV Cuda CM Saber R Turner JD Gierut AK Haines GK III. Nonclassical Ly6C(-) monocytes drive the development of inflammatory arthritis in mice. Cell Rep. (2014) 9:591–604. 10.1016/j.celrep.2014.09.032 25373902 PMC4223808

[203] Zheng Z Chiu S Akbarpour M Sun H Reyfman PA Anekalla KR. Donor pulmonary intravascular nonclassical monocytes recruit recipient neutrophils and mediate primary lung allograft dysfunction. Sci Transl Med. (2017) 9:eaal4508. 10.1126/scitranslmed.aal4508 28615357 PMC5568853

[204] Tatham KC O'dea KP Romano R Donaldson HE Wakabayashi K Patel BV. Intravascular donor monocytes play a central role in lung transplant ischaemia-reperfusion injury. Thorax. (2018) 73:350–60. 10.1136/thoraxjnl-2016-208977 28389600 PMC5870457

[205] Carlin LM Stamatiades EG Auffray C Hanna RN Glover L Vizcay-Barrena G. Nr4a1-dependent Ly6C(low) monocytes monitor endothelial cells and orchestrate their disposal. Cell. (2013) 153:362–75. 10.1016/j.cell.2013.03.010 23582326 PMC3898614

[206] Boyette LB Macedo C Hadi K Elinoff BD Walters JT Ramaswami B. Phenotype, function, and differentiation potential of human monocyte subsets. PLoS ONE. (2017) 12:e0176460. 10.1371/journal.pone.0176460 28445506 PMC5406034

[207] Patel AA Zhang Y Fullerton JN Boelen L Rongvaux A Maini AA. The fate and lifespan of human monocyte subsets in steady state and systemic inflammation. J Exp Med. (2017) 214:1913–23. 10.1084/jem.20170355 28606987 PMC5502436

[208] Kapellos TS Bonaguro L Gemünd I Reusch N Saglam A Hinkley ER. Human monocyte subsets and phenotypes in major chronic inflammatory diseases. Front Immunol. (2019) 10:2035. 10.3389/fimmu.2019.02035 31543877 PMC6728754

[209] Hashimoto D Chow A Noizat C Teo P Beasley MB Leboeuf M. Tissue-resident macrophages self-maintain locally throughout adult life with minimal contribution from circulating monocytes. Immunity. (2013) 38:792–804. 10.1016/j.immuni.2013.04.004 23601688 PMC3853406

[210] Seidler S Zimmermann HW Bartneck M Trautwein C Tacke F. Age-dependent alterations of monocyte subsets and monocyte-related chemokine pathways in healthy adults. BMC Immunol. (2010) 11:30. 10.1186/1471-2172-11-30 20565954 PMC2910032

[211] Costantini A Viola N Berretta A Galeazzi R Matacchione G Sabbatinelli J. Age-related M1/M2 phenotype changes in circulating monocytes from healthy/unhealthy individuals. Aging. (2018) 10:1268–80. 10.18632/aging.101465 29885276 PMC6046240

[212] Greiffo F Fernandez I Frankenberger M Behr J Eickelberg O Circulating monocytes from interstitial lung disease patients show an activated phenotype. Eur Respir J. (2016) 48:PA3894 10.1183/13993003.congress-2016.PA3894

[213] Cornwell WD Kim V Fan X Vega ME Ramsey FV Criner GJ. Activation and polarization of circulating monocytes in severe chronic obstructive pulmonary disease. BMC Pulm Med. (2018) 18:101. 10.1186/s12890-018-0664-y 29907106 PMC6003040

[214] Scott MKD Quinn K Li Q Carroll R Warsinske H Vallania F. Increased monocyte count as a cellular biomarker for poor outcomes in fibrotic diseases: a retrospective, multicentre cohort study. Lancet Respir Med. (2019) 7:497–508. 10.1016/S2213-2600(18)30508-3 30935881 PMC6529612

[215] Van Duin D Mohanty S Thomas V Ginter S Montgomery RR Fikrig E. Age-associated defect in human TLR-1/2 function. J Immunol. (2007) 178:970–5. 10.4049/jimmunol.178.2.970 17202359

[216] van Duin D Allore HG Mohanty S Ginter S Newman FK Belshe RB. Prevaccine determination of the expression of costimulatory B7 molecules in activated monocytes predicts influenza vaccine responses in young and older adults. J Infect Dis. (2007) 195:1590–7. 10.1086/516788 17471428

[217] Bessa-Gonçalves M Silva AM Brás JP Helmholz H Luthringer-Feyerabend BJC Willumeit-Römer R. Fibrinogen and magnesium combination biomaterials modulate macrophage phenotype, NF-kB signaling and crosstalk with mesenchymal stem/stromal cells. Acta Biomater. (2020) 114:471–84. 10.1016/j.actbio.2020.07.028 32688091

[218] Sylvestre M Crane CA Pun SH. Progress on modulating tumor-associated macrophages with biomaterials. Adv Mater. (2020) 32:e1902007. 10.1002/adma.201902007 31559665 PMC7098849

[219] Wofford KL Singh BS Cullen DK Spiller KL. Biomaterial-mediated reprogramming of monocytes via microparticle phagocytosis for sustained modulation of macrophage phenotype. Acta Biomater. (2020) 101:237–48. 10.1016/j.actbio.2019.11.021 31731024 PMC6960335

[220] Wright JR. Clearance and recycling of pulmonary surfactant. Am J Physiol. (1990) 259:L1–12. 10.1152/ajplung.1990.259.2.L1 2200279

[221] Soroosh P Doherty TA Duan W Mehta AK Choi H Adams YF. Lung-resident tissue macrophages generate Foxp3+ regulatory T cells and promote airway tolerance. J Exp Med. (2013) 210:775–88. 10.1084/jem.20121849 23547101 PMC3620360

[222] Westphalen K Gusarova GA Islam MN Subramanian M Cohen TS Prince AS. Sessile alveolar macrophages communicate with alveolar epithelium to modulate immunity. Nature. (2014) 506:503–6. 10.1038/nature12902 24463523 PMC4117212

[223] Ochs M Nyengaard JR Jung A Knudsen L Voigt M Wahlers T. The number of alveoli in the human lung. Am J Respir Crit Care Med. (2004) 169:120–4. 10.1164/rccm.200308-1107OC 14512270

[224] Hume PS Gibbings SL Jakubzick CV Tuder RM Curran-Everett D Henson PM. Localization of macrophages in the human lung via design-based stereology. Am J Respir Crit Care Med. (2020) 201:1209–17. 10.1164/rccm.201911-2105OC 32197050 PMC7233346

[225] Bharat A Bhorade SM Morales-Nebreda L Mcquattie-Pimentel AC Soberanes S Ridge K. Flow cytometry reveals similarities between lung macrophages in humans and mice. Am J Respir Cell Mol Biol. (2016) 54:147–9. 10.1165/rcmb.2015-0147LE 26274047 PMC4742931

[226] Guilliams M De Kleer I Henri S Post S Vanhoutte L De Prijck S. Alveolar macrophages develop from fetal monocytes that differentiate into long-lived cells in the first week of life via GM-CSF. J Exp Med. (2013) 210:1977. 10.1084/jem.20131199 24043763 PMC3782041

[227] Tan SY Krasnow MA. Developmental origin of lung macrophage diversity. Development. (2016) 143:1318–27. 10.1242/dev.129122 26952982 PMC4852511

[228] Stremmel C Schuchert R Wagner F Thaler R Weinberger T Pick R Yolk sac macrophage progenitors traffic to the embryo during defined stages of development. Nat Commun. (2018) 9:75 10.1038/s41467-018-06065-9 29311541 PMC5758709

[229] Misharin AV Morales-Nebreda L Mutlu GM Budinger GR Perlman H. Flow cytometric analysis of macrophages and dendritic cell subsets in the mouse lung. Am J Respir Cell Mol Biol. (2013) 49:503–10. 10.1165/rcmb.2013-0086MA 23672262 PMC3824047

[230] Evren E Ringqvist E Willinger T. Origin and ontogeny of lung macrophages: from mice to humans. Immunology. (2020) 160:126–38. 10.1111/imm.13154 31715003 PMC7218405

[231] Gibbings SL Thomas SM Atif SM Mccubbrey AL Desch AN Danhorn T. Three unique interstitial macrophages in the murine lung at steady state. Am J Respir Cell Mol Biol. (2017) 57:66–76. 10.1165/rcmb.2016-0361OC 28257233 PMC5516280

[232] Sabatel C Radermecker C Fievez L Paulissen G Chakarov S Fernandes C. Exposure to bacterial CpG DNA protects from airway allergic inflammation by expanding regulatory lung interstitial macrophages. Immunity. (2017) 46:457–73. 10.1016/j.immuni.2017.02.016 28329706

[233] Liegeois M Legrand C Desmet CJ Marichal T Bureau F. The interstitial macrophage: a long-neglected piece in the puzzle of lung immunity. Cell Immunol. (2018) 330:91–6. 10.1016/j.cellimm.2018.02.001 29458975

[234] Schyns J Bai Q Ruscitti C Radermecker C De Schepper S Chakarov S. Non-classical tissue monocytes and two functionally distinct populations of interstitial macrophages populate the mouse lung. Nat Commun. (2019) 10:3964. 10.1038/s41467-019-11843-0 31481690 PMC6722135

[235] Chakarov S Lim HY Tan L Lim SY See P Lum J. Two distinct interstitial macrophage populations coexist across tissues in specific subtissular niches. Science. (2019) 363:eaau0964. 10.1126/science.aau0964 30872492

[236] Ural BB Yeung ST Damani-Yokota P Devlin JC de Vries M Vera-Licona P. Identification of a nerve-associated, lung-resident interstitial macrophage subset with distinct localization and immunoregulatory properties. Sci Immunol. (2020) 5:eaax8756. 10.1126/sciimmunol.aax8756 32220976 PMC7717505

[237] Joshi N Watanabe S Verma R Jablonski RP Chen C-I Cheresh P. A spatially restricted fibrotic niche in pulmonary fibrosis is sustained by M-CSF/M-CSFR signalling in monocyte-derived alveolar macrophages. Eur Respir J. (2020) 55:1900646. 10.1183/13993003.00646-2019 31601718 PMC6962769

[238] Venosa A Malaviya R Choi H Gow AJ Laskin JD Laskin DL. Characterization of distinct macrophage subpopulations during nitrogen mustard-induced lung injury and fibrosis. Am J Respir Cell Mol Biol. (2016) 54:436–46. 10.1165/rcmb.2015-0120OC 26273949 PMC4821033

[239] Venosa A Smith LC Murray A Banota T Gow AJ Laskin JD. Regulation of macrophage foam cell formation during nitrogen mustard (NM)-induced pulmonary fibrosis by lung lipids. Toxicol Sci. (2019) 172:344–58. 10.1093/toxsci/kfz187 31428777 PMC6876262

[240] Smith LC Venosa A Gow AJ Laskin JD Laskin DL. Transcriptional profiling of lung macrophages during pulmonary injury induced by nitrogen mustard. Ann N Y Acad Sci. (2020). 10.1111/nyas.14444 32767459 PMC7901013

[241] Fry AM Shay DK Holman RC Curns AT Anderson LJ Trends in hospitalizations for pneumonia among persons aged 65 years or older in the United States, 1988-2002. JAMA. (2005) 294:2712–9. 10.1001/jama.294.21.2712 16333006

[242] Wong CK Smith CA Sakamoto K Kaminski N Koff JL Goldstein DR. Aging impairs alveolar macrophage phagocytosis and increases influenza-induced mortality in mice. J Immunol. (2017) 199:1060–8. 10.4049/jimmunol.1700397 28646038 PMC5557035

[243] Mcquattie-Pimentel AC Ren Z Joshi N Watanabe S Stoeger T Chi M The aging microenvironment shapes alveolar macrophage identity in aging. bioRxiv. (2019) 717033 10.1101/717033

[244] Libby DM. The Eosinophil in idiopathic pulmonary fibrosis. Chest. (1987) 92:7–8. 10.1378/chest.92.1.7 3595251

[245] Alberts WM. Eosinophilic interstitial lung disease. Curr Opin Pulm Med. (2004) 10:419–24. 10.1097/01.mcp.0000130330.29422.8d 15316442

[246] Birring SS Parker D Mckenna S Hargadon B Brightling CE Pavord ID. Sputum eosinophilia in idiopathic pulmonary fibrosis. Inflamm Res. (2005) 54:51–6. 10.1007/s00011-004-1321-x 15750711

[247] Aceves SS. Remodeling and fibrosis in chronic eosinophil inflammation. Dig Dis. (2014) 32:15–21. 10.1159/000357004 24603375 PMC4037288

[248] Barnes NC Sharma R Lettis S Calverley PMA. Blood eosinophils as a marker of response to inhaled corticosteroids in COPD. Eur Respir J. (2016) 47:1374–82. 10.1183/13993003.01370-2015 26917606

[249] Brix N Rasmussen F Poletti V Bendstrup E. Eosinophil alveolitis in two patients with idiopathic pulmonary fibrosis. Respir Med Case Rep. (2016) 19:61–4. 10.1016/j.rmcr.2016.07.010 27625983 PMC5010638

[250] Wen T Rothenberg ME. The regulatory function of eosinophils. Microbiol Spectr. (2016) 4:257–69. 10.1128/microbiolspec.MCHD-0020-2015 27780017 PMC5088784

[251] Acharya KR Ackerman SJ. Eosinophil granule proteins: form and function. J Biol Chem. (2014) 289:17406–15. 10.1074/jbc.R113.546218 24802755 PMC4067173

[252] Mesnil C Raulier S Paulissen G Xiao X Birrell MA Pirottin D. Lung-resident eosinophils represent a distinct regulatory eosinophil subset. J Clin Invest. (2016) 126:3279–95. 10.1172/JCI85664 27548519 PMC5004964

[253] Fujimoto K Kubo K Yamaguchi S Honda T Matsuzawa Y. Eosinophil activation in patients with pulmonary fibrosis. Chest. (1995) 108:48–54. 10.1378/chest.108.1.48 7606990

[254] Katz LE Gleich GJ Hartley BF Yancey SW Ortega HG. Blood eosinophil count is a useful biomarker to identify patients with severe eosinophilic asthma. Ann Am Thorac Soc. (2014) 11:531–6. 10.1513/AnnalsATS.201310-354OC 24606022

[255] Wu H-X Zhuo K-Q Cheng D-Y. Peripheral blood eosinophil as a biomarker in outcomes of acute exacerbation of chronic obstructive pulmonary disease. Int J Chron Obstruct Pulmon Dis. (2019) 14:3003–15. 10.2147/COPD.S226783 31920297 PMC6935282

[256] Idiopathic Pulmonary Fibrosis Clinical Research Network Raghu G Anstrom KJ King TE Jr Lasky JA Martinez FJ. Prednisone, azathioprine, and N-acetylcysteine for pulmonary fibrosis. N Engl J Med. (2012) 366:1968–77. 10.1056/NEJMoa1113354 22607134 PMC3422642

[257] Juarez MM Chan AL Norris AG Morrissey BM Albertson TE. Acute exacerbation of idiopathic pulmonary fibrosis-a review of current and novel pharmacotherapies. J Thorac Dis. (2015) 7:499–519. 10.3978/j.issn.2072-1439.2015.01.17 25922733 PMC4387423

[258] Wiertz I Wuyts W van Moorsel C Vorselaars R Ten Hoedt-Zijp M van Es W Negative outcome of prednisone in possible idiopathic pulmonary fibrosis. Eur Respir J. (2016) 48:OA4571 10.1183/13993003.congress-2016.OA4571

[259] Druilhe A Létuvé S Pretolani M. Glucocorticoid-induced apoptosis in human eosinophils: mechanisms of action. Apoptosis. (2003) 8:481–95. 10.1023/A:1025590308147 12975579

[260] Yagi T Sato A Hayakawa H Ide K. Failure of aged rats to accumulate eosinophils in allergic inflammation of the airway. J Allergy Clin Immunol. (1997) 99:38–47. 10.1016/S0091-6749(97)70298-7 9003209

[261] Thomas RA Green RH Brightling CE Birring SS Parker D Wardlaw AJ. The influence of age on induced sputum differential cell counts in normal subjects. Chest. (2004) 126:1811–4. 10.1016/S0012-3692(15)31427-6 15596678

[262] Mathur SK Schwantes EA Jarjour NN Busse WW. Age-related changes in eosinophil function in human subjects. Chest. (2008) 133:412–9. 10.1378/chest.07-2114 18252914 PMC2919352

[263] Jonas DE Wines RCM Delmonte M Amick HR Wilkins TM Einerson BD Drug class reviews. In: Drug Class Review: Controller Medications for Asthma: Final Update 1 Report. Portland, OR: Oregon Health and Science University Copyright © 2011 by Oregon Health and Science University) (2011). 22132427

[264] Bafadhel M Mckenna S Terry S Mistry V Pancholi M Venge P. Blood eosinophils to direct corticosteroid treatment of exacerbations of chronic obstructive pulmonary disease: a randomized placebo-controlled trial. Am J Respir Crit Care Med. (2012) 186:48–55. 10.1164/rccm.201108-1553OC 22447964 PMC3400995

[265] Pazdrak K Moon Y Straub C Stafford S Kurosky A. Eosinophil resistance to glucocorticoid-induced apoptosis is mediated by the transcription factor NFIL3. Apoptosis. (2016) 21:421–31. 10.1007/s10495-016-1226-5 26880402 PMC4769953

[266] Benson VS Pascoe KC Siddall J Small M Müllerová H. Exacerbation frequency and eosinophil counts among patients with COPD currently prescribed triple therapy. Int J Chron Obstruct Pulmon Dis. (2019) 14:2711–23. 10.2147/COPD.S217503 31819403 PMC6890196

[267] Brigger D Riether C van Brummelen R Mosher KI Shiu A Ding Z. Eosinophils regulate adipose tissue inflammation and sustain physical and immunological fitness in old age. Nat Metab. (2020) 2:688–702. 10.1038/s42255-020-0228-3 32694825 PMC7438316

[268] Kushwah R Hu J. Complexity of dendritic cell subsets and their function in the host immune system. Immunology. (2011) 133:409–19. 10.1111/j.1365-2567.2011.03457.x 21627652 PMC3143352

[269] Zareian N Aprile S Cristaldi L Ligotti ME Vasto S Farzaneh F. Triggering of toll-like receptors in old individuals. Relevance for vaccination. Curr Pharm Des. (2019) 25:4163–7. 10.2174/1381612825666191111155800 31713478

[270] Agrawal A Agrawal S Gupta S. Role of dendritic cells in inflammation and loss of tolerance in the elderly. Front Immunol. (2017) 8:896. 10.3389/fimmu.2017.00896 28798751 PMC5526855

[271] Brown CC Gudjonson H Pritykin Y Deep D Lavallée VP Mendoza A. Transcriptional basis of mouse and human dendritic cell heterogeneity. Cell. (2019) 179:846–63.e24. 10.1016/j.cell.2019.09.035 31668803 PMC6838684

[272] Kopf M Schneider C Nobs SP. The development and function of lung-resident macrophages and dendritic cells. Nat Immunol. (2015) 16:36–44. 10.1038/ni.3052 25521683

[273] Schraml BU van Blijswijk J Zelenay S Whitney PG Filby A Acton SE. Genetic tracing via DNGR-1 expression history defines dendritic cells as a hematopoietic lineage. Cell. (2013) 154:843–58. 10.1016/j.cell.2013.07.014 23953115

[274] Laouar Y Welte T Fu XY Flavell RA. STAT3 is required for Flt3L-dependent dendritic cell differentiation. Immunity. (2003) 19:903–12. 10.1016/S1074-7613(03)00332-7 14670306

[275] Sridharan A Esposo M Kaushal K Tay J Osann K Agrawal S. Age-associated impaired plasmacytoid dendritic cell functions lead to decreased CD4 and CD8 T cell immunity. Age. (2011) 33:363–76. 10.1007/s11357-010-9191-3 20953722 PMC3168606

[276] Mohamed EA Sayed WM. Implication of JAK1/STAT3/SOCS3 pathway in aging of cerebellum of male rat: histological and molecular study. Sci Rep. (2020) 10:8840. 10.1038/s41598-020-64050-z 32483368 PMC7264275

[277] Tort Tarrés M Maus R Stolper J Aschenbrenner F Welte T Gauldie J Role of dendritic cells in pulmonary fibrosis in mice. Eur Respir J. (2016) 48:PA3891 10.1183/13993003.congress-2016.PA3891

[278] Kato A Hulse KE Tan BK Schleimer RP. B-lymphocyte lineage cells and the respiratory system. J Allergy Clin Immunol. (2013) 131:933–58. 10.1016/j.jaci.2013.02.023 23540615 PMC3628816

[279] Stankovic B Bjørhovde HAK Skarshaug R Aamodt H Frafjord A Müller E. Immune cell composition in human non-small cell lung cancer. Front Immunol. (2019) 9:3101. 10.3389/fimmu.2018.03101 30774636 PMC6367276

[280] Montecino-Rodriguez E Leathers H Dorshkind K. Identification of a B-1 B cell-specified progenitor. Nat Immunol. (2006) 7:293–301. 10.1038/ni1301 16429139

[281] Ansel KM Harris RB Cyster JG. CXCL13 is required for B1 cell homing, natural antibody production, and body cavity immunity. Immunity. (2002) 16:67–76. 10.1016/S1074-7613(01)00257-6 11825566

[282] Baumgarth N. The double life of a B-1 cell: self-reactivity selects for protective effector functions. Nat Rev Immunol. (2011) 11:34–46. 10.1038/nri2901 21151033

[283] Xu Z Butfiloski EJ Sobel ES Morel L. Mechanisms of peritoneal B-1a cells accumulation induced by murine lupus susceptibility locus Sle2. J Immunol. (2004) 173:6050–8. 10.4049/jimmunol.173.10.6050 15528340

[284] Barlow JL Bellosi A Hardman CS Drynan LF Wong SH Cruickshank JP. Innate IL-13-producing nuocytes arise during allergic lung inflammation and contribute to airways hyperreactivity. J Allergy Clin Immunol. (2012) 129:191–8.e191–4. 10.1016/j.jaci.2011.09.041 22079492

[285] Diana J Simoni Y Furio L Beaudoin L Agerberth B Barrat F. Crosstalk between neutrophils, B-1a cells and plasmacytoid dendritic cells initiates autoimmune diabetes. Nat Med. (2013) 19:65–73. 10.1038/nm.3042 23242473

[286] Weng NP Granger L Hodes RJ. Telomere lengthening and telomerase activation during human B cell differentiation. Proc Natl Acad Sci USA. (1997) 94:10827–32. 10.1073/pnas.94.20.10827 9380719 PMC23499

[287] Hao Y O'neill P Naradikian MS Scholz JL Cancro MP. A B-cell subset uniquely responsive to innate stimuli accumulates in aged mice. Blood. (2011) 118:1294–304. 10.1182/blood-2011-01-330530 21562046 PMC3152496

[288] Ma S Wang C Mao X Hao Y. B cell dysfunction associated with aging and autoimmune diseases. Front Immunol. (2019) 10:318. 10.3389/fimmu.2019.00318 30873171 PMC6400972

[289] Stephan RP Reilly CR Witte PL. Impaired ability of bone marrow stromal cells to support B-lymphopoiesis with age. Blood. (1998) 91:75–88. 10.1182/blood.V91.1.75.75_75_88 9414271

[290] Russell Knode LM Naradikian MS Myles A Scholz JL Hao Y Liu D. Age-Associated B cells express a diverse repertoire of VH and Vkappa genes with somatic hypermutation. J Immunol. (2017) 198:1921–7. 10.4049/jimmunol.1601106 28093524 PMC5322232

[291] Rubtsov AV Rubtsova K Fischer A Meehan RT Gillis JZ Kappler JW. Toll-like receptor 7 (TLR7)-driven accumulation of a novel CD11c? B-cell population is important for the development of autoimmunity. Blood. (2011) 118:1305–15. 10.1182/blood-2011-01-331462 21543762 PMC3152497

[292] Polverino F Cosio BG Pons J Laucho-Contreras M Tejera P Iglesias A. B cell-activating factor. An orchestrator of lymphoid follicles in severe chronic obstructive pulmonary disease. Am J Respir Crit Care Med. (2015) 192:695–705. 10.1164/rccm.201501-0107OC 26073875 PMC4595676

[293] Morissette MC Gao Y Shen P Thayaparan D Bérubé JC Paré PD. Role of BAFF in pulmonary autoantibody responses induced by chronic cigarette smoke exposure in mice. Physiol Rep. (2016) 4:e13057. 10.14814/phy2.13057 28039405 PMC5210376

[294] Wallace WA Howie SE Krajewski AS Lamb D. The immunological architecture of B-lymphocyte aggregates in cryptogenic fibrosing alveolitis. J Pathol. (1996) 178:323–9. 8778339 10.1002/(SICI)1096-9896(199603)178:3<323::AID-PATH467>3.0.CO;2-7

[295] John-Schuster G Günter S Hager K Conlon TM Eickelberg O Yildirim AÖ. Inflammaging increases susceptibility to cigarette smoke-induced COPD. Oncotarget. (2016) 7:30068–83. 10.18632/oncotarget.4027 26284585 PMC5058664

[296] Tan H-X Esterbauer R Vanderven HA Juno JA Kent SJ Wheatley AK. Inducible bronchus-associated lymphoid tissues (iBALT) serve as sites of B cell selection and maturation following influenza infection in mice. Front Immunol. (2019) 10:611. 10.3389/fimmu.2019.00611 30984186 PMC6450362

[297] Komura K Yanaba K Horikawa M Ogawa F Fujimoto M Tedder TF. CD19 regulates the development of bleomycin-induced pulmonary fibrosis in a mouse model. Arthritis Rheum. (2008) 58:3574–84. 10.1002/art.23995 18975313

[298] François A Gombault A Villeret B Alsaleh G Fanny M Gasse P. B cell activating factor is central to bleomycin- and IL-17-mediated experimental pulmonary fibrosis. J Autoimmun. (2015) 56:1–11. 10.1016/j.jaut.2014.08.003 25441030

[299] Schiller HB Mayr CH Leuschner G Strunz M Staab-Weijnitz C Preisendörfer S. Deep proteome profiling reveals common prevalence of MZB1-positive plasma B cells in human lung and skin fibrosis. Am J Respir Crit Care Med. (2017) 196:1298–310. 10.1164/rccm.201611-2263OC 28654764 PMC6913086

[300] Heukels P van Hulst JAC van Nimwegen M Boorsma CE Melgert BN. Enhanced Bruton's tyrosine kinase in B-cells and autoreactive IgA in patients with idiopathic pulmonary fibrosis. Respir Res. (2019) 20:232. 10.1186/s12931-019-1195-7 31651327 PMC6814043

[301] Dunn RM Busse PJ Wechsler ME. Asthma in the elderly and late-onset adult asthma. Allergy. (2018) 73:284–94. 10.1111/all.13258 28722758

[302] Holodick NE Rodríguez-Zhurbenko N Hernández AM. Defining natural antibodies. Front Immunol. (2017) 8:872. 10.3389/fimmu.2017.00872 28798747 PMC5526850

[303] Prata L Ovsyannikova IG Tchkonia T Kirkland JL. Senescent cell clearance by the immune system: emerging therapeutic opportunities. Semin Immunol. (2018) 40:101275. 10.1016/j.smim.2019.04.003 31088710 PMC7061456

[304] Chou C Li MO. Tissue-resident lymphocytes across innate and adaptive lineages. Front Immunol. (2018) 9:2104. 10.3389/fimmu.2018.02104 30298068 PMC6160555

[305] Ardain A Marakalala MJ Leslie A. Tissue-resident innate immunity in the lung. Immunology. (2020) 159:245–56. 10.1111/imm.13143 31670391 PMC7011639

[306] Kaech SM Cui W. Transcriptional control of effector and memory CD8+ T cell differentiation. Nat Rev Immunol. (2012) 12:749–61. 10.1038/nri3307 23080391 PMC4137483

[307] Fan X Rudensky AY. Hallmarks of tissue-resident lymphocytes. Cell. (2016) 164:1198–211. 10.1016/j.cell.2016.02.048 26967286 PMC4973889

[308] Golubovskaya V Wu L. Different subsets of T cells, memory, effector functions, and CAR-T immunotherapy. Cancers. (2016) 8:36. 10.3390/cancers8030036 26999211 PMC4810120

[309] Lei L Zhao C Qin F He ZY Wang X Zhong XN. Th17 cells and IL-17 promote the skin and lung inflammation and fibrosis process in a bleomycin-induced murine model of systemic sclerosis. Clin Exp Rheumatol. (2016) 34(Suppl. 100):14–22. 26750756

[310] Zhu X Cui J Yi L Qin J Tulake W Teng F. The role of T cells and macrophages in asthma pathogenesis: a new perspective on mutual crosstalk. Mediat Inflamm. (2020) 2020:7835284. 10.1155/2020/7835284 32922208 PMC7453253

[311] Kim HR Hwang KA Park SH Kang I. IL-7 and IL-15: biology and roles in T-Cell immunity in health and disease. Crit Rev Immunol. (2008) 28:325–39. 10.1615/CritRevImmunol.v28.i4.40 19166383

[312] Vadasz Z Haj T Kessel A Toubi E. Age-related autoimmunity. BMC Med. (2013) 11:94. 10.1186/1741-7015-11-94 23556986 PMC3616810

[313] Pizzolla A Nguyen THO Sant S Jaffar J Loudovaris T Mannering SI. Influenza-specific lung-resident memory T cells are proliferative and polyfunctional and maintain diverse TCR profiles. J Clin Invest. (2018) 128:721–33. 10.1172/JCI96957 29309047 PMC5785253

[314] Strutt TM Dhume K Finn CM Hwang JH Castonguay C Swain SL. IL-15 supports the generation of protective lung-resident memory CD4 T cells. Mucosal Immunol. (2018) 11:668–80. 10.1038/mi.2017.101 29186108 PMC5975122

[315] Chou JP Effros RB. T cell replicative senescence in human aging. Curr Pharm Des. (2013) 19:1680–98. 10.2174/138161213805219711 23061726 PMC3749774

[316] Xu W Larbi A. Markers of T cell senescence in humans. Int J Mol Sci. (2017) 18:1742. 10.3390/ijms18081742 28796199 PMC5578132

[317] Pangrazzi L Weinberger B. T cells, aging and senescence. Exp Gerontol. (2020) 134:110887. 10.1016/j.exger.2020.110887 32092501

[318] Youn J-C Jung MK Yu HT Kwon J-S Kwak J-E Park S-H. Increased frequency of CD4+CD57+ senescent T cells in patients with newly diagnosed acute heart failure: exploring new pathogenic mechanisms with clinical relevance. Sci Rep. (2019) 9:12887. 10.1038/s41598-019-49332-5 31501486 PMC6733929

[319] Tserel L Kolde R Limbach M Tretyakov K Kasela S Kisand K. Age-related profiling of DNA methylation in CD8+ T cells reveals changes in immune response and transcriptional regulator genes. Sci Rep. (2015) 5:13107. 10.1038/srep13107 26286994 PMC4541364

[320] Goronzy JJ Hu B Kim C Jadhav RR Weyand CM. Epigenetics of T cell aging. J Leukoc Biol. (2018) 104:691–9. 10.1002/JLB.1RI0418-160R 29947427 PMC6162101

[321] Dejaco C Duftner C Schirmer M. Are regulatory T-cells linked with aging? Exp Gerontol. (2006) 41:339–45. 10.1016/j.exger.2006.01.008 16516426

[322] Schmitt V Rink L Uciechowski P. The Th17/Treg balance is disturbed during aging. Exp Gerontol. (2013) 48:1379–86. 10.1016/j.exger.2013.09.003 24055797

[323] Yasuda K Takeuchi Y Hirota K The pathogenicity of Th17 cells in autoimmune diseases. Semin Immunopathol. (2019) 41:283–97. 10.1007/s00281-019-00733-8 30891627

[324] Jing Y Gravenstein S Chaganty NR Chen N Lyerly KH Joyce S. Aging is associated with a rapid decline in frequency, alterations in subset composition, and enhanced Th2 response in CD1d-restricted NKT cells from human peripheral blood. Exp Gerontol. (2007) 42:719–32. 10.1016/j.exger.2007.01.009 17368996

[325] Novak J Dobrovolny J Novakova L Kozak T. The decrease in number and change in phenotype of mucosal-associated invariant T cells in the elderly and differences in men and women of reproductive age. Scand J Immunol. (2014) 80:271–5. 10.1111/sji.12193 24846411

[326] Rodriguez-Garcia M Fortier JM Barr FD Wira CR. Aging impacts CD103(+) CD8(+) T cell presence and induction by dendritic cells in the genital tract. Aging Cell. (2018) 17:e12733. 10.1111/acel.12733 29455474 PMC5946085

[327] Lee OJ Cho YN Kee SJ Kim MJ Jin HM Lee SJ. Circulating mucosal-associated invariant T cell levels and their cytokine levels in healthy adults. Exp Gerontol. (2014) 49:47–54. 10.1016/j.exger.2013.11.003 24269212

[328] Wong EB Ndung'u T Kasprowicz VO. The role of mucosal-associated invariant T cells in infectious diseases. Immunology. (2017) 150:45–54. 10.1111/imm.12673 27633333 PMC5341498

[329] Collins SL Chan-Li Y Oh M Vigeland CL Limjunyawong N Mitzner W. Vaccinia vaccine-based immunotherapy arrests and reverses established pulmonary fibrosis. JCI Insight. (2016) 1:e83116. 10.1172/jci.insight.83116 27158671 PMC4855513

[330] Hegde P Weiss E Paradis V Wan J Mabire M Sukriti S. Mucosal-associated invariant T cells are a profibrogenic immune cell population in the liver. Nat Commun. (2018) 9:2146. 10.1038/s41467-018-04450-y 29858567 PMC5984626

[331] Law BMP Wilkinson R Wang X Kildey K Giuliani K Beagley KW. Human tissue-resident mucosal-associated invariant T (MAIT) Cells in renal fibrosis and CKD. J Am Soc Nephrol. (2019) 30:1322–35. 10.1681/ASN.2018101064 31186283 PMC6622420

[332] Lezmi G Leite-De-Moraes M. Invariant natural killer T and mucosal-associated invariant T cells in asthmatic patients. Front Immunol. (2018) 9:1766. 10.3389/fimmu.2018.01766 30105031 PMC6077286

[333] Faunce DE Palmer JL Paskowicz KK Witte PL Kovacs EJ. CD1d-restricted NKT cells contribute to the age-associated decline of T cell immunity. J Immunol. (2005) 175:3102–9. 10.4049/jimmunol.175.5.3102 16116199

[334] Hansen MJ Chan SP Langenbach SY Dousha LF Jones JE Yatmaz S. IL-17A and serum amyloid A are elevated in a cigarette smoke cessation model associated with the persistence of pigmented macrophages, neutrophils and activated NK cells. PLoS ONE. (2014) 9:e113180. 10.1371/journal.pone.0113180 25405776 PMC4236152

[335] Pichavant M Rémy G Bekaert S Le Rouzic O Kervoaze G Vilain E. Oxidative stress-mediated iNKT-cell activation is involved in COPD pathogenesis. Mucosal Immunol. (2014) 7:568–78. 10.1038/mi.2013.75 24172846 PMC3998637

[336] Baker DJ Wijshake T Tchkonia T Lebrasseur NK Childs BG van de Sluis B. Clearance of p16Ink4a-positive senescent cells delays ageing-associated disorders. Nature. (2011) 479:232–6. 10.1038/nature10600 22048312 PMC3468323

[337] Ustarroz-Cano M García-Peláez I Piñón-Zárate G Herrera-Enríquez M Soldevila G Fortoul TI. CD11c decrease in mouse thymic dendritic cells after vanadium inhalation. J Immunotoxicol. (2012) 9:374–80. 10.3109/1547691X.2012.673181 22512508

[338] Hong F Zhou Y Zhou Y Wang L. Immunotoxic effects of thymus in mice following exposure to nanoparticulate TiO(2). Environ Toxicol. (2017) 32:2234–43. 10.1002/tox.22439 28646487

[339] Yi H-S Kim SY Kim JT Lee Y-S Moon JS Kim M. T-cell senescence contributes to abnormal glucose homeostasis in humans and mice. Cell Death Dis. (2019) 10:249. 10.1038/s41419-019-1494-4 30867412 PMC6416326

[340] Baskara I Kerbrat S Dagouassat M Nguyen HQ Guillot-Delost M Surenaud M. Cigarette smoking induces human CCR6+Th17 lymphocytes senescence and VEGF-A secretion. Sci Rep. (2020) 10:6488. 10.1038/s41598-020-63613-4 32300208 PMC7162978

[341] Martos SN Campbell MR Lozoya OA Wang X Bennett BD Thompson IJB Single-cell analyses identify dysfunctional CD16+ CD8 T cells in smokers. Cell Rep Med. (2020) 1:100054 10.1016/j.xcrm.2020.100054 33163982 PMC7644053

[342] Hams E Bermingham R Fallon PG. Macrophage and innate lymphoid cell interplay in the genesis of fibrosis. Front Immunol. (2015) 6:597. 10.3389/fimmu.2015.00597 26635811 PMC4655423

[343] Mikami Y Takada Y Hagihara Y Kanai T. Innate lymphoid cells in organ fibrosis. Cytok Growth Factor Rev. (2018) 42:27–36. 10.1016/j.cytogfr.2018.07.002 30104153

[344] D'souza SS Shen X Fung ITH Ye L Kuentzel M Chittur SV. Compartmentalized effects of aging on group 2 innate lymphoid cell development and function. Aging Cell. (2019) 18:e13019. 10.1111/acel.13019 31429526 PMC6826140

[345] Fung ITH Sankar P Zhang Y Robison LS Zhao X D'souza SS. Activation of group 2 innate lymphoid cells alleviates aging-associated cognitive decline. J Exp Med. (2020) 217:e20190915. 10.1084/jem.20190915 32022838 PMC7144523

[346] de Gonzalo-Calvo D Neitzert K Fernández M Vega-Naredo I Caballero B García-Macía M. Differential inflammatory responses in aging and disease: TNF-alpha and IL-6 as possible biomarkers. Free Radic Biol Med. (2010) 49:733–7. 10.1016/j.freeradbiomed.2010.05.019 20639132

[347] Drakopanagiotakis F Wujak L Wygrecka M Markart P. Biomarkers in idiopathic pulmonary fibrosis. Matrix Biol. (2018) 68–69:404–21. 10.1016/j.matbio.2018.01.023 29408012

[348] Ortiz-Quintero B Buendía-Roldán I Ramírez-Salazar EG Balderas-Martínez YI Ramírez-Rodríguez SL Martínez-Espinosa K. Circulating microRNA signature associated to interstitial lung abnormalities in respiratory asymptomatic subjects. Cells. (2020) 9:1556. 10.3390/cells9061556 32604783 PMC7348836

[349] Hannum G Guinney J Zhao L Zhang L Hughes G Sadda S. Genome-wide methylation profiles reveal quantitative views of human aging rates. Mol Cell. (2013) 49:359–67. 10.1016/j.molcel.2012.10.016 23177740 PMC3780611

[350] Conforti F Davies ER Calderwood CJ Thatcher TH Jones MG Smart DE. The histone deacetylase inhibitor, romidepsin, as a potential treatment for pulmonary fibrosis. Oncotarget. (2017) 8:48737–54. 10.18632/oncotarget.17114 28467787 PMC5564721

[351] Lee JU Son JH Shim EY Cheong HS Shin SW Shin HD. Global DNA methylation pattern of fibroblasts in idiopathic pulmonary fibrosis. DNA Cell Biol. (2019) 38:905–14. 10.1089/dna.2018.4557 31305135

[352] Huan T Chen G Liu C Bhattacharya A Rong J Chen BH. Age-associated microRNA expression in human peripheral blood is associated with all-cause mortality and age-related traits. Aging Cell. (2018) 17:e12687. 10.1111/acel.12687 29044988 PMC5770777

[353] Aiello A Farzaneh F Candore G Caruso C Davinelli S Gambino CM Immunosenescence and its hallmarks: how to oppose aging strategically? A review of potential options for therapeutic intervention. Front Immunol. (2019) 10:2247 10.3389/fimmu.2019.02247 31608061 PMC6773825

[354] Ozsvari B Nuttall JR Sotgia F Lisanti MP Azithromycin and Roxithromycin define a new family of “senolytic” drugs that target senescent human fibroblasts. Aging. (2018) 10:3294–307. 10.18632/aging.101633 30428454 PMC6286845

[355] Garg G Singh S Singh AK Rizvi SI. Antiaging effect of metformin on brain in naturally aged and accelerated senescence model of rat. Rejuv Res. (2017) 20:173–82. 10.1089/rej.2016.1883 27897089

[356] Cork GK Thompson J Slawson C. Real talk: the inter-play between the mTOR, AMPK, and hexosamine biosynthetic pathways in cell signaling. Front Endocrinol. (2018) 9:522. 10.3389/fendo.2018.00522 30237786 PMC6136272

[357] Weichhart T. mTOR as regulator of lifespan, aging, and cellular senescence: a mini-review. Gerontology. (2018) 64:127–34. 10.1159/000484629 29190625 PMC6089343

[358] Malavolta M Bracci M Santarelli L Sayeed MA Pierpaoli E Giacconi R. Inducers of senescence, toxic compounds, and senolytics: the multiple faces of Nrf2-activating phytochemicals in cancer adjuvant therapy. Mediators Inflamm. (2018) 2018:4159013. 10.1155/2018/4159013 29618945 PMC5829354

[359] Rangarajan S Bone NB Zmijewska AA Jiang S Park DW Bernard K Metformin reverses established lung fibrosis in a bleomycin model. Nat Med. (2018) 24:1121–7. 10.1038/s41591-018-0087-6 29967351 PMC6081262

[360] Hohmann MS Habiel DM Coelho AL Verri WA Jr Hogaboam CM. Quercetin enhances ligand-induced apoptosis in senescent idiopathic pulmonary fibrosis fibroblasts and reduces lung fibrosis *in vivo*. Am J Respir Cell Mol Biol. (2019) 60:28–40. 10.1165/rcmb.2017-0289OC 30109946 PMC6348716

[361] Justice JN Nambiar AM Tchkonia T Lebrasseur NK Pascual R Hashmi SK. Senolytics in idiopathic pulmonary fibrosis: results from a first-in-human, open-label, pilot study. EBioMedicine. (2019) 40:554–63. 10.1016/j.ebiom.2018.12.052 30616998 PMC6412088

[362] Kheirollahi V Wasnick RM Biasin V Vazquez-Armendariz AI Chu X Moiseenko A. Metformin induces lipogenic differentiation in myofibroblasts to reverse lung fibrosis. Nat Commun. (2019) 10:2987. 10.1038/s41467-019-10839-0 31278260 PMC6611870

[363] Golpanian S El-Khorazaty J Mendizabal A Difede DL Suncion VY Karantalis V. Effect of aging on human mesenchymal stem cell therapy in ischemic cardiomyopathy patients. J Am Coll Cardiol. (2015) 65:125–32. 10.1016/j.jacc.2014.10.040 25593053 PMC4405121

[364] Schulman IH Balkan W Hare JM. Mesenchymal stem cell therapy for aging frailty. Front Nutr. (2018) 5:108–108. 10.3389/fnut.2018.00108 30498696 PMC6249304

[365] Harrell CR Sadikot R Pascual J Fellabaum C Jankovic MG Jovicic N. Mesenchymal stem cell-based therapy of inflammatory lung diseases: current understanding and future perspectives. Stem Cells Int. (2019) 2019:4236973. 10.1155/2019/4236973 31191672 PMC6525794

[366] Caplan AI Correa D. The MSC: an injury drugstore. Cell Stem Cell. (2011) 9:11–5. 10.1016/j.stem.2011.06.008 21726829 PMC3144500

[367] Roved J Westerdahl H Hasselquist D. Sex differences in immune responses: hormonal effects, antagonistic selection, and evolutionary consequences. Horm Behav. (2017) 88:95–105. 10.1016/j.yhbeh.2016.11.017 27956226

[368] Márquez EJ Chung C-H Marches R Rossi RJ Nehar-Belaid D Eroglu A. Sexual-dimorphism in human immune system aging. Nat Commun. (2020) 11:751. 10.1038/s41467-020-14396-9 32029736 PMC7005316

